# Human HLA-DR^+^CD27^+^ regulatory T cells show enhanced antigen-specific suppressive function

**DOI:** 10.1172/jci.insight.162978

**Published:** 2023-12-08

**Authors:** Xiaoqian Ma, Lu Cao, Martina Raneri, Hannah Wang, Qi Cao, Yuanfei Zhao, Naiara G. Bediaga, Gaetano Naselli, Leonard C. Harrison, Wayne J. Hawthorne, Min Hu, Shounan Yi, Philip J. O’Connell

**Affiliations:** 1 Centre for Transplantation and Renal Research, Westmead Institute for Medical Research, University of Sydney, Sydney, New South Wales, Australia.; 2 Cell Transplantation and Gene Therapy Institute, The Third Xiangya Hospital, Central South University, Changsha, China.; 3 Walter and Eliza Hall Institute of Medical Research, University of Melbourne, Melbourne, Victoria, Australia.

**Keywords:** Immunology, Transplantation, Immunotherapy, T cells, Tolerance

## Abstract

Regulatory T cells (Tregs) have potential for the treatment of autoimmune diseases and graft rejection. Antigen specificity and functional stability are considered critical for their therapeutic efficacy. In this study, expansion of human Tregs in the presence of porcine PBMCs (xenoantigen-expanded Tregs, Xn-Treg) allowed the selection of a distinct Treg subset, coexpressing the activation/memory surface markers HLA-DR and CD27 with enhanced proportion of FOXP3^+^Helios^+^ Tregs. Compared with their unsorted and HLA-DR^+^CD27^+^ double-positive (DP) cell–depleted Xn-Treg counterparts, HLA-DR^+^CD27^+^ DP-enriched Xn-Tregs expressed upregulated Treg function markers CD95 and ICOS with enhanced suppression of xenogeneic but not polyclonal mixed lymphocyte reaction. They also had less Treg-specific demethylation in the region of FOXP3 and were more resistant to conversion to effector cells under inflammatory conditions. Adoptive transfer of porcine islet recipient NOD/SCID IL2 receptor γ^–/–^ mice with HLA-DR^+^CD27^+^ DP-enriched Xn-Tregs in a humanized mouse model inhibited porcine islet graft rejection mediated by 25-fold more human effector cells. The prolonged graft survival was associated with enhanced accumulation of FOXP3^+^ Tregs and upregulated expression of Treg functional genes, IL10 and cytotoxic T lymphocyte antigen 4, but downregulated expression of effector Th1, Th2, and Th17 cytokine genes, within surviving grafts. Collectively, human HLA-DR^+^CD27^+^ DP-enriched Xn-Tregs expressed a specific regulatory signature that enabled identification and isolation of antigen-specific and functionally stable Tregs with potential as a Treg-based therapy.

## Graphical abstract

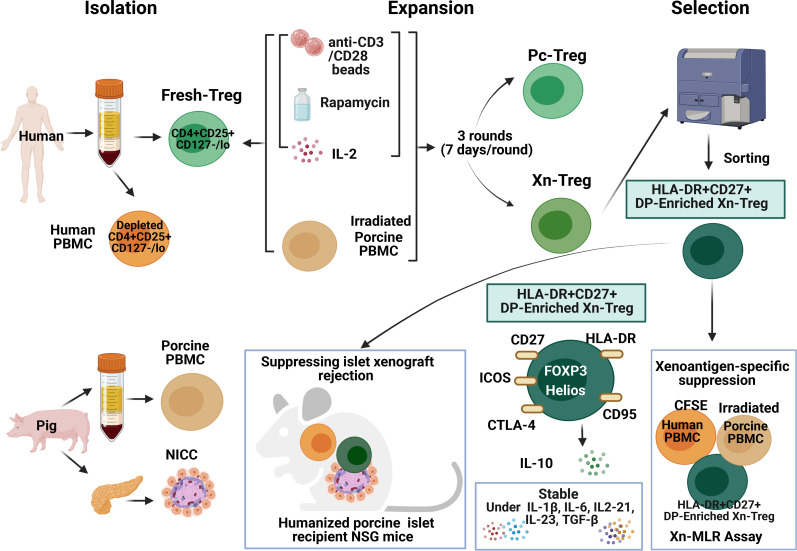

## Introduction

Regulatory T cells (Tregs) are an essential component of immune homeostasis. Tregs were initially characterized as CD4^+^CD25^+^ T cells in mouse models of autoimmune disease and make up 5% to 10% of peripheral CD4^+^ T cells (1, 2). FOXP3 is the key transcription factor that characterizes this population of thymically derived Tregs (2, 3). In humans, mutation of the *FOXP3* gene leads to severe autoimmune disease, demonstrating the importance of this cell subset in suppressing unwanted inflammatory responses to self-antigens (4). In addition to their role in preventing autoimmunity, CD4^+^CD25^+^ T cells that express FOXP3 have been shown to be important for suppressing alloimmunity and for inducing and maintaining allograft tolerance (5, 6). In models of transplantation, dominant tolerance is characterized by indefinite graft survival after an initial brief period of induction therapy in the presence of an otherwise intact immune system. In other words, this nonresponsiveness is antigen specific. Given their potential to reduce the requirement for immunosuppression, CD4^+^CD25^+^FOXP3^+^ Tregs have been pursued for their therapeutic potential to suppress autoimmunity and to reduce or eliminate the requirement for immunosuppression after transplantation. Trials of naive Tregs in kidney or liver transplantation have been disappointing in that the results have been modest and the relatively large number of Tregs required has provided regulatory, cost, and production challenges (7–9).

Animal studies of transplant tolerance have shown that both the induction and maintenance of tolerance depend on the development of antigen-specific Tregs, as deletion of Foxp3^+^ Tregs leads to prompt rejection (10, 11). Also, a relatively small number of cells can transfer graft-specific tolerance to a naive host, as has been shown by numerous adoptive transfer studies (12–14). The observations are equally true for autoimmunity as they are for all immunity. For example, in models of autoimmune type 1 diabetes, antigen-specific Tregs, which were isolated from pancreatic lymph nodes or pulsed with islet antigen, were superior to polyclonal Tregs at preventing or curing the disease (15, 16). Similarly, alloantigen-specific Tregs, enriched by alloantigen-stimulated expansion in vitro or engineered to express a T cell receptor (TCR) transgene, were more effective than polyclonal Tregs at preventing rejection of organ and tissue grafts. Studies in humanized mouse models have shown similar results: alloantigen-expanded human Tregs were more potent suppressors of skin graft rejection than were their polyclonal counterparts (14), and human alloantigen-specific Tregs generated with an HLA-A2–specific chimeric antigen receptor were superior to polyclonal Tregs at preventing xenogeneic graft versus host disease (GVHD) caused by HLA-A2^+^ T cells (17). An important advantage of enhanced Treg antigen specificity is that the suppression is targeted to the graft, hence avoiding potential opportunistic infection and malignancy that may result from nonspecific suppression as a result of the application of polyclonal Tregs.

One of the problems in developing in vitro–expanded Tregs has been identifying markers that would facilitate their selection after stimulation. Intracellular FOXP3 expression, enhanced Helios expression, and demethylation of a Treg-specific demethylated region (TSDR) within the FOXP3 locus represent the gold standard for estimating the fraction of stable Tregs within a population. However, it does not allow for sorting a specific subset that could be used therapeutically. The lack of discriminative markers also affects systematic functional optimization of in vitro–generated Tregs, such as genetically engineered Tregs with transgenic TCR or chimeric antigen receptor (CAR) constructs. To identify the characteristics of an antigen-activated subset, we have used a model of Treg development to xenoantigens such as porcine PBMC or neonatal porcine islet cell clusters (NICCs) (18, 19). Due to the phylogenetic distance between pig and humans, these provide a large antigen load and hence provide a larger pool of antigen-specific Tregs available for study. For instance, we have also reported that human Tregs expanded ex vivo with xenoantigen stimulation are more potent than polyclonal Tregs at suppressing xenoreactive effector cell proliferation in a xenogeneic mixed lymphocyte reaction (MLR), although they are equally suppressive as polyclonal Tregs in an allogeneic or polyclonal MLR xenogeneic response, indicating acquisition of xenoantigen specificity after xenoantigen stimulation (18).

A number of Treg activation-induced surface markers, such as HLA-DR (20), CD27 (21), CD45RO (22), and ICOS (23), have been described to identify activated and/or memory Tregs (24). Among those, HLA-DR^+^ Tregs have been reported to express higher levels of Treg-associated activation markers and produce lower levels of effector cytokines (20, 24). HLA-DR^+^ Tregs are present in human peripheral blood, thymus, and umbilical cord blood and are more suppressive than HLA-DR^–^ Tregs in vitro (20). CD27 expression has also been associated with increased Treg suppressive function (21, 25). It has been shown that a CD4^+^CD127^−/lo^CD25^+^CD45RA^−^ Treg subpopulation cultured in vitro with tacrolimus lost their Treg TSDR demethylation phenotype, which correlated with a reduction of CD27 expression, suggesting an association of CD27 expression with Treg stability (26). Thus, given that human Tregs expanded with xenoantigen have been demonstrated already to express upregulated levels of activated/effector markers, HLA-DR, ICOS, and CD45RO, which were associated with their enhanced suppression (18), it is feasible that one or a combination of surface activation markers could be identified as a specific signature for the isolation of a stable, antigen-specific Treg subset that could be suitable for clinical immunotherapy.

Based on previous findings, we wished to test the hypothesis that human Tregs expanded in the presence of antigen and expressing the surface markers of HLA-DR and CD27 would represent a stable antigen-specific Treg population that is more potent than their unsorted counterparts at suppressing a graft immune response. To test this hypothesis, we used the human T cell response to porcine PBMCs in vitro and NICCs in vivo as a proof-of-concept study, where a human HLA-DR^+^CD27^+^ double-positive (DP) Treg subset from xenoantigen-expanded Tregs was compared in efficacy and stability with their unsorted, HLA-DR^+^CD27^+^ DP-depleted Treg and polyclonal Treg counterparts at protecting islet xenografts from rejection mediated by human T cells.

## Results

### Xenoantigen-expanded Tregs express upregulated levels of Treg activation/memory markers HLA-DR and CD27.

**Figure 1:**
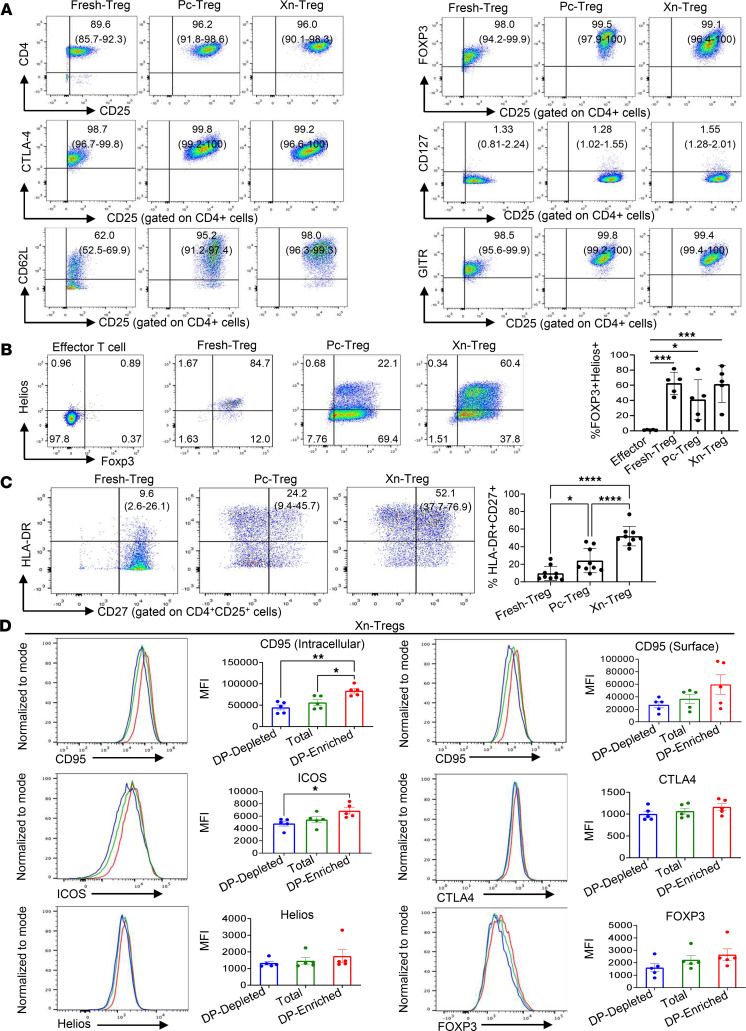
Phenotypical characterization of ex vivo–expanded human Tregs. Representative flow cytometric plots of CD4^+^CD25^+^CD127^–/lo^ Treg phenotypes isolated from human PBMCs (Fresh-Treg), Tregs expanded with anti-CD3/CD28 dynabeads (Pc-Treg), and stimulation in presence of irradiated porcine PBMCs (Xn-Treg) after 3 cycles (weeks) of stimulation. (**A**) Gates were set on CD4^+^ T cells. FOXP3 and other cell surface marker expression shown as the percentage of CD4^+^ T cells coexpressing individual Treg markers (CD4^+^CD25^+^, CD25^+^FOXP3^+^, CD25^+^CTLA4^+^, CD127^–^CD25^+^, CD62L^+^CD25^+^, CD25^+^GITR^+^). (**B**) The proportion of Tregs coexpressing FOXP3 and Helios on Fresh-Tregs, Pc-Tregs, Xn-Tregs and negative control effector T cells. The gating strategies are shown in Supplemental Figure 1. (**C**) The proportion of Tregs coexpressing HLA-DR and CD27 after gating on CD4^+^CD25^+^ cells. (**D**) Phenotyping of Xn-Tregs. Representative histograms of CD95 expression (surface and intracellular staining), ICOS (surface staining), CTLA4 (surface staining), FOXP3 (intracellular staining), and Helios (intracellular staining) on HLA-DR^+^CD27^+^ double-positive enriched Xn-Treg (DP-enriched; red line), total Xn-Treg (Total; green line), and Xn-Treg depleted of HLA-DR^+^CD27^+^ double positive cells (DP-depleted; blue line). Expression of CD95, ICOS, CTLA4, FOXP3, and Helios in different Treg subsets is also shown by the mean fluorescence intensity (MFI). Numbers in brackets in each plot are the ranges of the percentage of individual Treg markers detected in 4 independent experiments with Tregs from 4 individual donors (**A**) and 5 individual donors (**B**) and 7 independent experiments with 9 individual donors (**C**). Data represent 4 independent experiments with Xn-Tregs from 5 individual donors (**D**). Error bars indicate the mean ± SD (**A**–**C**) and mean ± SEM (**D**). One-way ANOVA: *P < 0.05, **P < 0.01, ***P < 0.001, and ****P < 0.0001.

Tregs (CD4^+^CD25^+^CD127^−/lo^) were isolated from human PBMCs and stimulated either with anti-CD3/CD28 beads alone (polyclonally stimulated Tregs, Pc-Treg) or with anti-CD3/CD28 dynabeads combined with irradiated porcine PBMCs (xenoantigen-expanded Tregs, Xn-Treg) for 3 rounds (7 d/round). Consistent with our previous study (18), Xn-Treg demonstrated similar levels of purity to their freshly isolated Treg (Fresh-Treg) and Pc-Treg counterparts (96% vs. 96.2% vs. 89.6% of CD4^+^CD25^+^ cells of Xn-Treg vs. Pc-Treg vs. Fresh-Treg), with high-level expression of FOXP3, cytotoxic T lymphocyte antigen 4 (CTLA4), glucocorticoid-induced tumor necrosis factor receptor (GITR), and CD62L (Figure 1A). In addition to their upregulated expression of the activation marker CD62L (98.0% vs. 62.0% of CD4^+^CD25^+^CD62L^+^ of Xn-Treg vs. Fresh-Treg), there was no change in CD127 expression between the 2 ex vivo–expanded Treg subsets and Fresh-Treg (Figure 1A). Next, the transcription factor Helios, a marker for Treg stability (27), and coexpression of FOXP3 were assessed. A similar proportion of FOXP3^+^Helios^+^ Tregs was seen in Xn-Treg (61.3% ± 24.3%) compared to Fresh-Treg (62.5% ± 14.7%), while Pc-Treg showed a decreased proportion of FOXP3^+^Helios^+^ Tregs (41.1% ± 26.3%) (Figure 1B and Supplemental Figure 1; supplemental material available online with this article; https://doi.org/10.1172/jci.insight.162978DS1), suggesting that Xn-Treg was a more stable Treg subset than Pc-Treg. The Xn-Treg also expressed higher levels of the Treg activation/memory surface markers HLA-DR and CD27 than PC-Treg, as evidenced by mean fluorescence intensity (MFI) (HLA-DR: 5,848 vs. 3,242.8 and CD27: 6,447.8 vs. 2,415.4 of Xn-Treg vs. Pc-Treg), while both types of expanded Tregs had similar levels of CD25 (MFI: 6,996 vs. 8,513.4 of Xn-Treg vs. Pc-Treg) and FOXP3 expression (MFI: 2,489.8 vs. 2,589 of Xn-Treg vs. Pc-Treg) (Supplemental Figure 2). Therefore, Xn-Treg showed a greater proportion of cells coexpressing HLA-DR and CD27 when compared with freshly isolated and polyclonally expanded Tregs (52% ± 11.0% vs. 24.3% ± 13.8% vs. 9.6% ± 8.0% of Xn-Treg vs. Pc-Treg vs. Fresh-Treg) (Figure 1C), suggesting that a Treg subset coexpressing HLA-DR and CD27 was selectively enriched in Xn-Treg.

We further analyzed the phenotype of the HLA-DR^+^CD27^+^ DP Treg subset of Xn-Treg (DP-enriched Xn-Treg) by flow cytometry, demonstrating that significantly enhanced intracellular expression of Treg function molecule CD95 (Fas) (28) was detected in HLA-DR^+^CD27^+^ DP-enriched Xn-Treg compared with either HLA-DR^+^CD27^+^ DP-depleted Xn-Treg or total Xn-Treg, as evidenced by MFI (83,708.2 ± 5,328.8 vs. 44,843.2 ± 5,879.9 vs. 56,270.4 ± 6,725.8 of DP-enriched Xn-Treg vs. DP-depleted Xn-Treg vs. total Xn-Treg) (Figure 1D). The enhanced CD95 expression was associated with enhanced HLA-DR and CD27 expression (Supplemental Figure 3A). Moreover, HLA-DR^+^CD27^+^ DP-enriched Xn-Treg expressed a significantly higher level of the Treg activation marker ICOS than DP-depleted Xn-Treg (MFI: 6,854.4 ± 498.5 vs. 4,790 ± 393.6 of DP-enriched Xn-Treg vs. DP-depleted Xn-Treg), and there was an increasing trend of ICOS expression when compared with total Xn-Treg (Figure 1D). The enhanced ICOS was also associated with HLA-DR expression (Supplemental Figure 3B). Although not significant, higher expression levels of the Treg activation marker CTLA4 (24), and the transcription factors, Helios and FOXP3, were seen in HLA-DR^+^CD27^+^ DP-enriched Xn-Treg, when compared with DP-depleted Xn-Treg or total Xn-Treg (Figure 1D). Furthermore, HLA-DR^+^CD27^+^ DP-enriched Xn-Treg had significantly increased FOXP3 expression when compared with either HLA-DR^–^CD27^–^ or HLA-DR^+^CD27^–^ Xn-Treg subsets, indicating enhanced FOXP3 expression tracked with CD27 expression (Supplemental Figure 3C).

**Figure 2:**
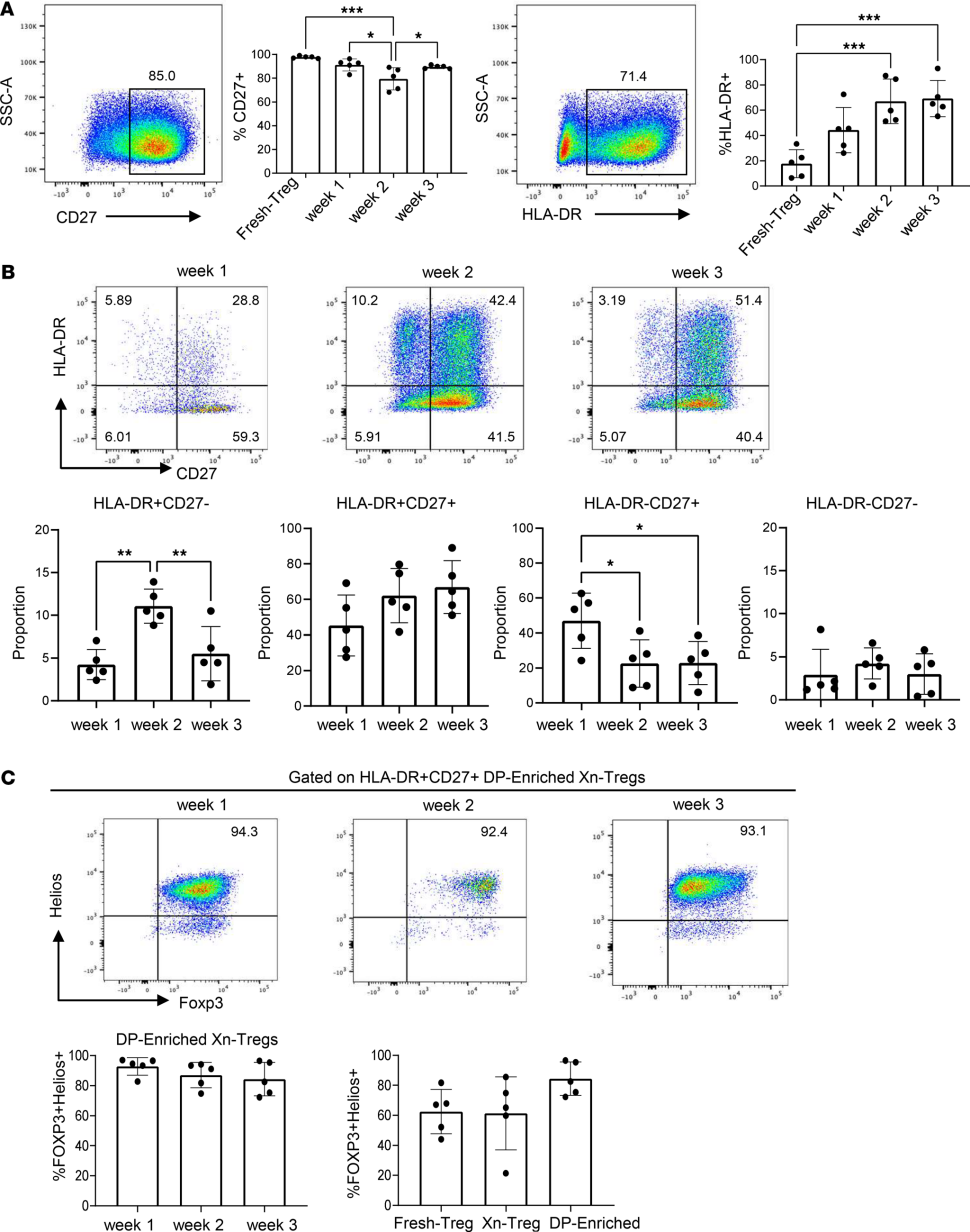
HLA-DR and/or CD27 expression within Xn-Tregs at different stimulation times and their FOXP3 and Helios expression. (**A**) The proportion of Xn-Tregs expressing HLA-DR or CD27 after gating on CD4^+^ cells after round (week) 1, 2, and 3 of stimulation. (**B**) The proportions of HLA-DR^+^CD27^–^, HLA-DR^+^CD27^+^, HLA-DR^–^CD27^+^, HLA-DR^–^CD27^–^ subsets within Xn-Tregs (after gating on CD4^+^ cells) following round 1, 2, and 3 of stimulation. The numbers in the corners represent the percentage of cells in each quadrant. (**C**) The representative flow cytometric plots and the percentage of FOXP3^+^Helios^+^ cells on HLA-DR^+^CD27^+^ DP-enriched Xn-Tregs following rounds 1, 2, and 3 of stimulation (after gating on HLA-DR^+^CD27^+^ cells) and the proportion of FOXP3^+^Helios^+^ cells in Fresh-Tregs, total Xn-Tregs, and HLA-DR^+^CD27^+^ DP-enriched Xn-Tregs. Data represent 3 independent experiments with Treg from 5 individual donors. Error bars indicate the mean ± SD. One-way ANOVA: *P < 0.05, **P < 0.01, and ***P < 0.001.

Next, we explored the expression of CD27 and HLA-DR within Xn-Treg across different stages of expansion. For the first 2 rounds of stimulation, the proportion of CD27^+^ Xn-Treg was decreased slightly when compared with Fresh-Treg. However, by round 3 of stimulation, the proportion of CD27^+^ Xn-Treg was increased (compared with round 2) (Figure 2A). By contrast, the proportion of HLA-DR^+^ Xn-Treg increased incrementally after each round of stimulation (Figure 2A). As a consequence, coexpression of CD27 and HLA-DR increased from round 1 to round 3 of stimulation, while the other 3 subsets either decreased (HLA-DR^–^CD27^+^ subset) or remained unchanged (HLA-DR^+^CD27^–^ and HLA-DR^–^CD27^–^ subsets) (Figure 2B). Consistent with this observation, CD27^+^HLA-DR^+^ DP-enriched Xn-Treg had a higher proportion of FOXP3^+^Helios^+^ cells than total Xn-Treg or Fresh-Treg, and this higher proportion of FOXP3^+^Helios^+^ cells was observed across different expansion stages, suggesting stability of the HLA-DR^+^CD27^+^ DP-enriched Xn-Treg subset (Figure 2C). Collectively, these findings suggested that 3 rounds of stimulation and expansion in the presence of antigen resulted in a highly stable HLA-DR^+^CD27^+^ DP-enriched Xn-Treg subset.

### The HLA-DR^+^CD27^+^ DP-enriched Xn-Treg subset is more suppressive and xenoantigen specific.

**Figure 3:**
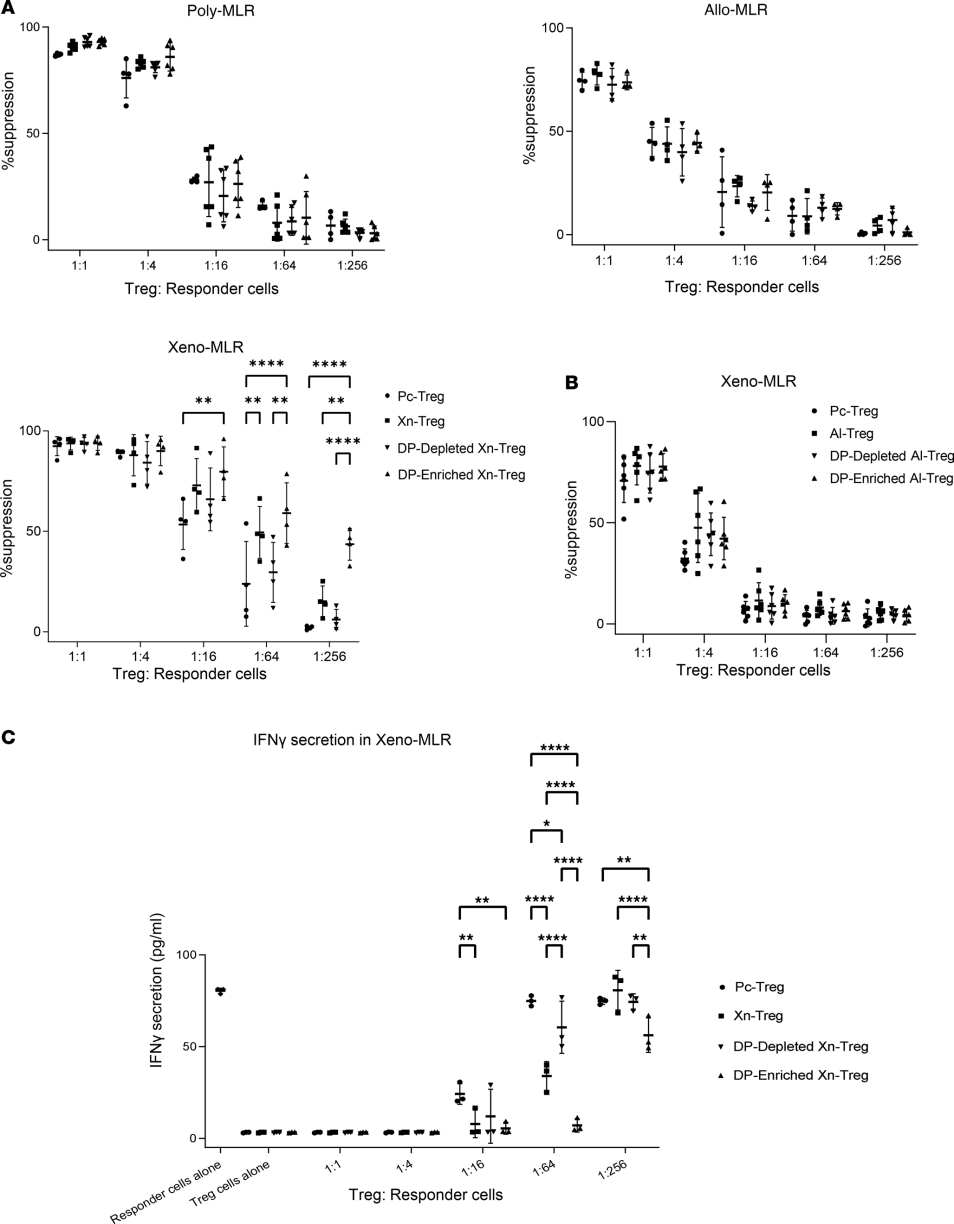
In vitro suppression assay of HLA-DR^+^CD27^+^ DP-enriched Xn-Tregs. (**A**) MLR assay of Xn-Treg suppressive capacity compared with Pc-Treg. Carboxyfluorescein diacetate succinimidyl ester–labeled (CFSE-labeled) autologous human PBMCs (CD4^+^CD25^+^CD127^–/lo^ depleted) were stimulated with irradiated xenogeneic porcine (Xn-MLR) or allogeneic (Allo MLR) PBMCs or anti-CD3/CD28 dynabeads (Poly MLR), in the presence or absence of serial dilutions of unsorted Xn-Treg or HLA-DR^+^CD27^+^ DP-enriched Xn-Treg or HLA-DR^+^CD27^+^ DP-depleted Xn-Treg or anti-CD3/CD28 dynabead–expanded Pc-Treg for 7 days, prior to measurement of PBMC proliferation by CFSE dilution. (**B**) Alloantigen-expanded Treg (Al-Treg) suppressive capacity in Xn-MLR. CFSE-labeled autologous human PBMCs were stimulated with irradiated xenogeneic porcine PBMCs, in the presence or absence of serial dilutions of unsorted Al-Treg or HLA-DR^+^CD27^+^ DP-enriched Al-Treg or HLA-DR^+^CD27^+^ DP-depleted Al-Treg or Pc-Treg for 7 days, prior to measurement of PBMC proliferation by CFSE dilution. (**C**) Assessment of IFN-γ concentration in supernatants of the Xn-MLR assay of Xn-Treg suppression. IFN-γ secretion in supernatants collected from xenogeneic MLR assay as described in **A** was measured by ELISA. Data are presented as mean ± SD of 4 independent experiments with Tregs from 4 individual donors (**A**) except in Poly MLR with 6 individual donors for Xn-Treg, DP-depleted Xn-Treg, and DP-enriched Xn-Treg, from 3 individual donors (**C**) and 6 independent experiments with Treg from 6 individual donors (**B**). Two-way ANOVA: *P < 0.05; **P < 0.01; ***P < 0.001; ****P < 0.0001.

To test this hypothesis, we undertook cell sorting to isolate HLA-DR^+^CD27^+^ Tregs from Xn-Tregs. Xn-Tregs were sorted into HLA-DR^+^CD27^+^ DP-enriched Xn-Treg and DP-depleted Xn-Treg subsets after undergoing a series of sequential cell gating (Supplemental Figure 4). Treg suppressive capacity was then assessed by MLR. Effector cells were stimulated with irradiated xenogeneic or allogeneic PBMCs or with polyclonally anti-CD3/CD28 dynabeads for xenogeneic, allogeneic, or polyclonal MLR assay, respectively. Tregs were added to the assay at predetermined suppressor-to-effector ratios to determine their efficacy at suppressing the respective MLRs. All Treg subsets tested, including Pc-Treg, unsorted Xn-Treg, DP-depleted Xn-Treg, and HLA-DR^+^CD27^+^ DP-enriched Xn-Treg, showed similar potency in inhibition of polyclonal or allostimulated MLRs in a Treg number-dependent manner (Figure 3A). However, consistent with our previous study (18), unsorted Xn-Tregs showed stronger suppressive capacity in the xenostimulated (Xeno) MLR than Pc-Tregs at lower Treg/responder ratios of 1:16 through 1:256, and this stronger potency in suppressing the xenogeneic but not polyclonal or allogeneic response was further enhanced by replacing unsorted Xn-Tregs with the sorted HLA-DR^+^CD27^+^ DP-enriched Xn-Treg subset in the Xeno MLR, showing that even at the lowest Treg/responder ratio tested (1:256), a 43.5% suppression of xenoreactive cell proliferation was still detected, which was not seen with other Treg subsets (43.5% vs. 1.97% vs. 15.1% vs. 6% of suppression by HLA-DR^+^CD27^+^ DP-enriched Xn-Treg vs. Pc-Treg vs. total Xn-Treg vs. DP-depleted Xn-Treg) (Figure 3A). The higher suppressive potency and xenoantigen stimulation-dependent suppression by HLA-DR^+^CD27^+^ DP-enriched Xn-Tregs was verified by depletion of HLA-DR^+^CD27^+^ DP cells from Xn-Tregs resulting in impaired Treg suppressive capacity and xenoantigen specificity as assessed by the Xeno MLR (Figure 3A). Using the same protocol of Xn-Treg expansion, alloantigen-expanded Tregs (Al-Treg) were generated from CD4^+^CD25^+^CD127^–/lo^ Tregs isolated from PBMCs stimulated with irradiated alloantigen PBMCs for 3 rounds. HLA-DR^+^CD27^+^ DP-enriched Al-Treg was equally suppressive of the Xeno MLR as HLA-DR^+^CD27^+^ DP-depleted Al-Treg or total Al-Treg, thereby suggesting that the antigen specificity and enhanced suppression were features of their xenoantigen stimulation and not solely the result of HLA-DR^+^ and CD27 coexpression (Figure 3B).

This finding was consistent with ICOS expression in the different expanded Treg populations. ICOS expression in HLA-DR^+^CD27^+^ DP-enriched Xn-Treg was significantly higher than that seen in DP-depleted Xn-Treg (*P* < 0.05) (Figure 1D), whereas there was no significant difference in ICOS expression in HLA-DR^+^CD27^+^ DP-enriched Pc-Treg (2,248 ± 772.9), HLA-DR^+^CD27^+^ DP-depleted Pc-Treg (1,686.4 ± 288.8), and total Pc-Treg (1,960.8 ± 551.2) (Supplemental Figure 5). This suggests that enhanced ICOS expression in HLA-DR^+^CD27^+^ DP-enriched Xn-Treg was a feature of xenoantigen simulation.

In addition to their higher capacity to specifically suppress the proliferating xenoreactive effector cells, HLA-DR^+^CD27^+^ DP-enriched Xn-Tregs were also more capable of suppressing IFN-γ secretion in the Xeno MLR cultures, where the biggest reduction in IFN-γ secretion was in the presence of HLA-DR^+^CD27^+^ DP-enriched Xn-Tregs even at the lower Treg/responder ratios of 1:16 through 1:256 (Figure 3C). Together, these results demonstrated that the HLA-DR^+^CD27^+^ DP-enriched Xn-Treg subset led to increased suppression of the xenogeneic response, including xenoreactive cell proliferation and effector cytokine secretion, and this suppression was antigen specific. Alternatively, depletion of HLA-DR^+^CD27^+^ cells from Xn-Tregs impaired their suppressive capacity and antigen specificity in vitro.

### HLA-DR^+^CD27^+^ DP-enriched Xn-Tregs are functionally stable in vitro.

**Figure 4:**
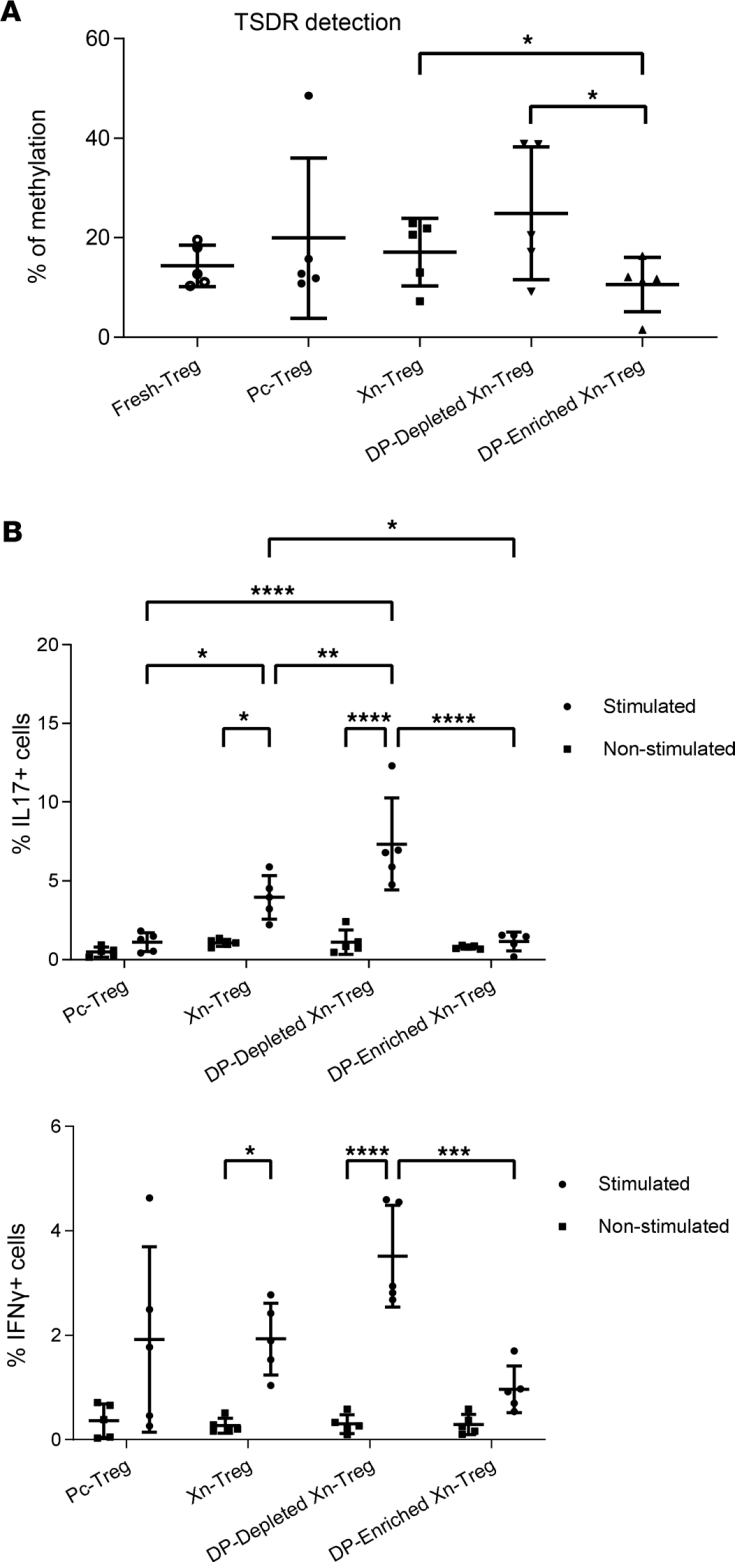
Evaluation of Treg functional stability. (**A**) TSDR assay. The stability of Treg master function marker FOXP3 was evaluated by measurement of the status of demethylation of TSDR within FOXP3 in all Treg subsets examined. Data are mean ± SD of independent experiments with Tregs from 5 individual donors. (**B**) Test of Treg plasticity and stability under pro-inflammatory conditions. The multiple types of Tregs were stimulated with a combination of pro-inflammatory cytokines (IL1β, IL6, IL21, IL23, TGF-β) and IL2 for 6 days (Stimulated) (details in Methods) prior to flow cytometric analysis of proportions of these cultured Tregs coexpressing IL17 or IFN-γ. Control samples were Treg subsets with IL2 only (nonstimulated). Data presented are mean ± SD of 5 independent experiments with Tregs from 5 individual donors. Paired *t* test comparison (2 tailed) between DP-enriched Xn-Treg and DP-depleted Xn-Treg or Xn-Treg (**A**) and 2-way ANOVA (**B**): *P < 0.05, **P < 0.01, ***P < 0.001, and ****P < 0.0001.

Since stable FOXP3 expression reflected by demethylation of TSDR within the FOXP3 gene is a prerequisite for the suppressive function, the functional stability of the HLA-DR^+^CD27^+^ DP-enriched Xn-Treg subset was evaluated by TSDR assay. The results showed that HLA-DR^+^CD27^+^ DP-enriched Xn-Treg retained a demethylation phenotype with no significant difference when compared to Fresh-Treg (10.6% ± 4.9% vs. 14.4% ± 3.7% of methylation in HLA-DR^+^CD27^+^ DP-enriched Xn-Treg vs. Fresh-Treg) and were also less methylated within their FOXP3 gene than total Xn-Treg and DP-depleted Xn-Treg counterparts, (10.6% ± 4.9% vs. 17.1% ± 6.1% vs. 24.9% ± 11.9% of methylation in HLA-DR^+^CD27^+^ DP-enriched Xn-Treg vs. total Xn-Treg vs. DP-depleted Xn-Treg) (Figure 4A). This verifies that the demethylation of the FOXP3 gene in expanded HLA-DR^+^CD27^+^ DP-enriched Xn-Tregs was similar to that of fresh, naive/rested, unexpanded Treg. HLA-DR^+^CD27^+^ DP-enriched Xn-Tregs are functionally stable and unlikely to revert to T effector cells. The functional stability of HLA-DR^+^CD27^+^ DP-enriched Xn-Tregs was further assessed under pro-inflammatory conditions to test their plasticity toward an effector Th17 or Th1 cell phenotype. Tregs were stimulated with a combination of pro-inflammatory cytokines for 6 days prior to detecting the proportion of CD4^+^FOXP3^+^ Tregs coexpressing IL-17 or IFN-γ. After stimulation, no significant change in proportion of IL-17–coexpressing cells was observed within both HLA-DR^+^CD27^+^ DP-enriched Xn-Tregs and Pc-Tregs (Figure 4B and Supplemental Figure 6). In contrast, a considerably increased proportion of IL-17–coexpressing cells was detected after inflammatory stimulation within total Xn-Tregs or DP-depleted Xn-Tregs, showing a significant difference from that seen within HLA-DR^+^CD27^+^DP-enriched Xn-Tregs (Figure 4B). Moreover, while all other Treg subsets tested demonstrated substantially increased IFN-γ–coexpressing cells upon inflammatory stimulation, HLA-DR^+^CD27^+^ DP-enriched Xn-Tregs showed a significantly reduced response to the stimulation, with a slight increase in IFN-γ–coexpressing cells (Figure 4B and Supplemental Figure 6). Taken together, these results indicated that HLA-DR^+^CD27^+^ DP-enriched Xn-Tregs were functionally stable in association with the expression of both HLA-DR and CD27 activation/memory markers.

### HLA-DR^+^CD27^+^ DP-enriched Xn-Tregs were more capable of suppressing islet xenograft rejection.

**Figure 5:**
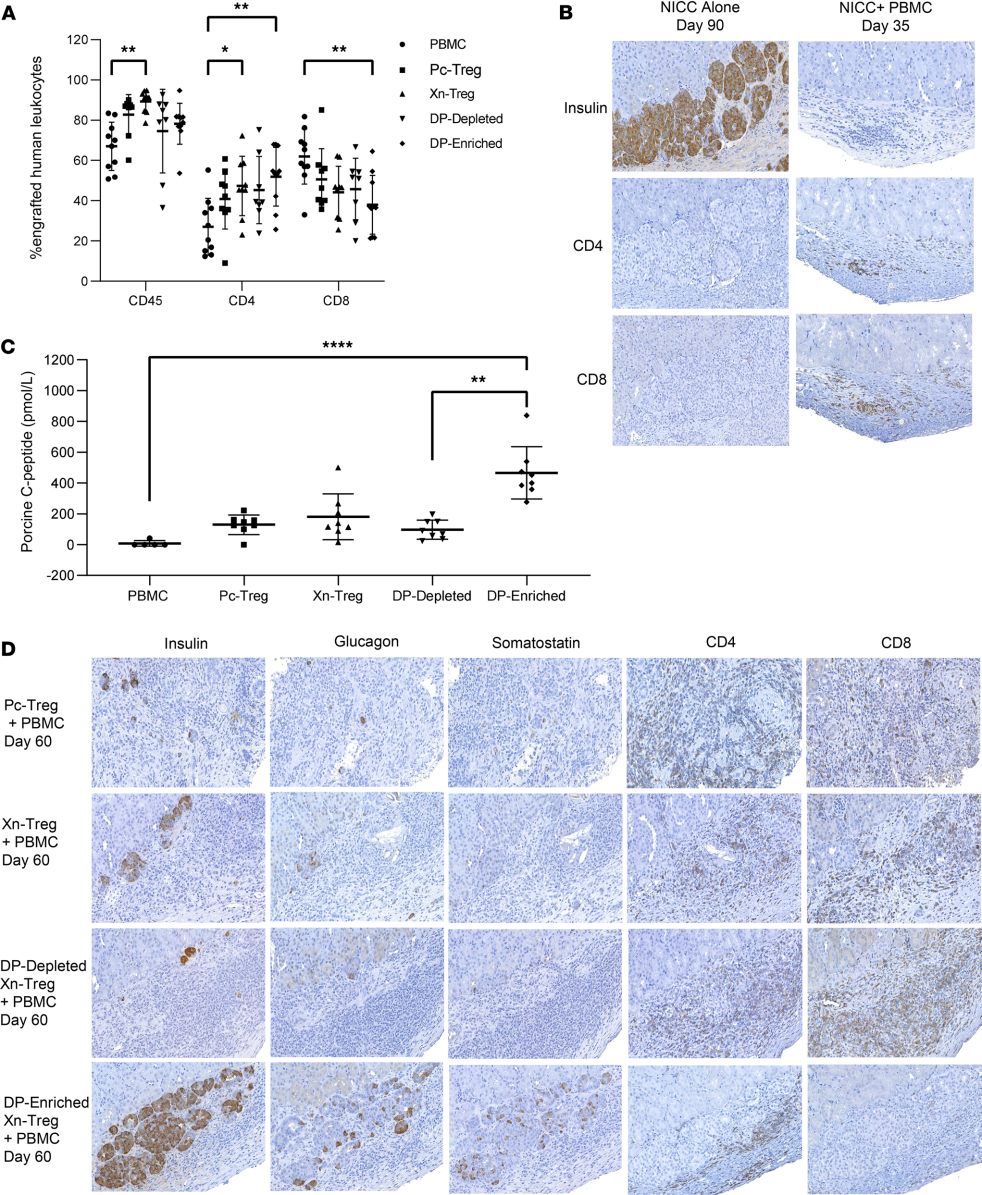
Evaluation of NICC xenograft survival and function in vivo. HLA-DR^+^CD27^+^ DP-enriched Xn-Tregs are more capable of suppressing islet xenograft injection. (**A**) Flow cytometric measurement of percentage of human leukocytes (CD45^+^ cells, CD4^+^ and CD8^+^ T cells) in the spleen of mice receiving human PBMCs (CD4^+^CD25^+^CD127^–/lo^ depleted) alone at 35 days, and different types of human expanded Tregs combined with PBMCs at 60 days after human cell transfer. Data are shown as mean ± SD of at least 3 independent experiments (*n* ≥ 8 mice of each group). (**B**) Representative immunohistochemical examination for human CD4 and CD8 and porcine insulin of graft samples from mice receiving no cells (NICC alone day 90 posttransplantation) or human PBMCs (NICC + PBMC day 35 after NICC transplantation). Original magnification, ×200. (**C**) Porcine C-peptide was measured at day 60 posttransplantation and control PBMC group at day 30. Data are represented as mean ± SD (*n* ≥ 8 mice of each group, except control PBMC group with *n* = 5). (**D**) Representative immunohistochemical staining of graft samples from the same mice as in **A** for human CD4 and CD8 and porcine insulin, glucagon, and somatostatin. Original magnification, ×200. One-way ANOVA with Tukey’s multiple-comparison test (**A**) and Kruskal-Wallis test (**C**): *P < 0.05; **P < 0.01; ****P < 0.0001.

To study HLA-DR^+^CD27^+^ DP-enriched Xn-Treg function in vivo, NOD/SCID IL-2 receptor γ^–/–^ (NSG) mice were transplanted with NICC xenografts and, 3 days after transplantation, reconstituted with or without 10^7^ human PBMCs (depleted of CD4^+^CD25^+^CD127^–/lo^ Tregs) as indicated in Supplemental Figure 7. Human PBMC engraftment was verified by flow cytometry at week 5 after islet transplantation, with 67.0% ± 11.9% of cells in spleen being human CD45^+^ cells, 27.1% ± 14.1 % CD4^+^ T cells, and 61.9% ± 13.7% CD8^+^ T cells (Figure 5A). While NICC grafts survived for at least 90 days in non-reconstituted recipients, mice with adoptively transferred engrafted human PBMCs rejected their xenografts completely within 35 days, with no visible insulin-positive-staining cells in the rejecting xenografts, compared with intact and insulin-positive-staining NICC grafts detected in non-reconstituted recipients (Figure 5B). Rejection was verified by porcine C-peptide assay with no detectable porcine C-peptide in reconstituted NICC graft recipients (<10 pmol/L) (Figure 5C). A large infiltrate of human CD4^+^ and CD8^+^ T cells was detected in the grafts of human PBMC-reconstituted recipients at the graft rejection time point (Figure 5B), similar to that seen in our previous study (29). Next, we tested the in vivo suppressive capability of the 3 groups of ex vivo–expanded human Treg subsets. NSG mice were transplanted with NICC xenografts and 3 days later injected with 1 × 10^7^ PBMCs (CD4^+^CD25^+^CD127^–/lo^ Treg depleted) and 4 × 10^5^ of the different Treg subsets at a ratio of PBMCs/Tregs of 25:1 (Supplemental Figure 7). All 3 groups of recipient mice demonstrated a similar large number of human CD45^+^ cells and CD4^+^ and CD8^+^ T cells in mouse spleen determined by flow cytometry 60 days after transfer (Figure 5A). This verified successful human leukocyte engraftment in these recipient mice, thereby ensuring that graft survival in the following experiments was the result of Treg-mediated suppression and not a failure of human leukocyte engraftment. In our previous study we showed adoptive transfer of NICC xenograft recipient mice with 2 × 10^6^ polyclonally expanded human Tregs was sufficient to suppress rejection induced by 1 × 10^7^ (5:1 of PBMCs/Tregs) human PBMC, with NICC xenograft surviving beyond 100 days after human PBMC rechallenge (29). In the study reported here, transplanted mice, adoptively transferred with 5-fold fewer Pc-Tregs (4 × 10^5^ at a ratio of 25:1 of PBMCs/Tregs), could not prevent NICC xenograft rejection, with all grafts rejected by day 60 after human cell transfer. Graft histology showed only a few single insulin-positive cells, with a large infiltrate of human CD4^+^ and CD8^+^ T cells (Figure 5D). Reconstitution of mice with human PBMCs and Xn-Tregs at the same ratio of PBMCs/Tregs (25:1) resulted in a little better islet xenograft survival, with the presence of small islet clusters with insulin- and glucagon-positive staining within a large infiltrate of human T cells 60 days after human cell transfer (Figure 5D). However, DP-depleted Xn-Treg were unable to achieve the same NICC xenograft survival as that shown by their Xn-Treg counterparts. When cotransferred at the same ratio of PBMCs, NICC grafts were rejected in a similar fashion to that found in PBMC and Pc-Treg cotransferred mice (Figure 5D). These results demonstrated that adoptive transfer to NICC recipients of nonselected Tregs at a ratio of 25:1 of PBMCs/Tregs was not sufficient or potent enough to effectively protect against human PBMC–mediated islet xenograft rejection in this model. In contrast, mice cotransferred with human PBMCs and HLA-DR^+^CD27^+^ DP-enriched Xn-Tregs at the same ratio of 25:1 prolonged NICC xenograft survival to at least 60 days after human cell transfer, as shown by intact surviving grafts containing endocrine-secreting cells including insulin-, glucagon-, and somatostatin-positive-staining cells, which were surrounded but not infiltrated by a small number of human CD4^+^ and CD8^+^ T cells (Figure 5D). This suggests that HLA-DR^+^CD27^+^ DP-enriched Xn-Tregs with xenoantigen specificity were substantially more potent at suppressing the xenogeneic response mediated by human PBMCs, thereby leading to superior islet xenograft survival in recipient mice. Graft survival beyond 60 days was difficult to assess as mice transferred with HLA-DR^+^CD27^+^ DP-enriched Xn-Tregs succumbed to GVHD beyond 60 days with intact NICC grafts, which again validated the relative antigen specificity for the anti-pig T cell response. In the 1 surviving recipient mouse the graft remained intact at day 90 after cell transfer (data not shown). This suggests that this protection mediated by HLA-DR^+^CD27^+^ DP-enriched Tregs was xenoantigen specific as it was protective of NICC xenograft rejection but not protective of human to mouse GVHD. To verify these histological findings, the in vivo function of the surviving grafts was further verified by porcine C-peptide assay. Consistent with the immunohistochemical findings, the highest levels of porcine C-peptide were detected in the serum from HLA-DR^+^CD27^+^ Treg cotransferred mice (465.5 ± 170.1 pmol/L) 60 days after human cell transfer compared with that seen in mice cotransferred with Xn-Treg (180.7± 149.6 pmol/L) or DP-depleted Xn-Tregs (97.1 ± 61.9 pmol/L) or Pc-Tregs (129.5± 63.7 pmol/L) (Figure 5C). Similar to the immunohistochemistry results there was no significant difference in porcine C-peptide levels among mice cotransferred with Pc-Treg or Xn-Treg or DP-depleted Xn-Treg subsets (Figure 5C).

### HLA-DR^+^CD27^+^ Tregs were able to selectively migrate to the graft after adoptive transfer to NSG recipients of NICC xenografts.

**Figure 6:**
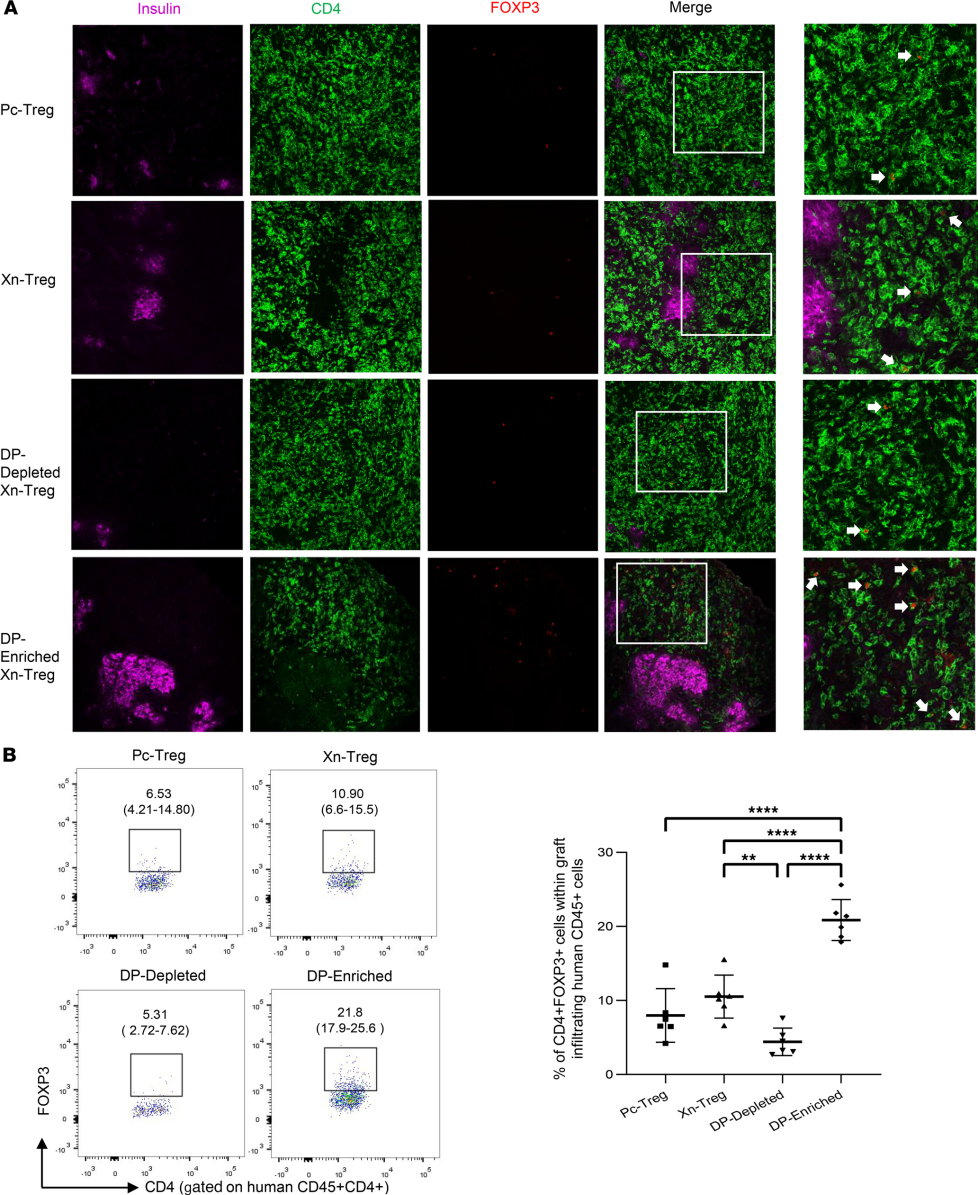
The graft-infiltrating CD4^+^FOXP3^+^ Treg. (**A**) Representative triple immunofluorescence staining for human CD4 and FOXP3 and porcine insulin of graft samples. NICC xenografts from mice receiving human PBMCs and different individual Treg subsets, at day 60 after human cell transfer, were stained for human CD4 in green, FOXP3 in red, and porcine insulin in purple. Original magnification, ×200 or ×400. (**B**) Representative flow cytometric plots and the proportion of CD4^+^FOXP3^+^ cells within graft-infiltrating human CD45^+^CD4^+^ cells by flow cytometric analysis. Data are the mean ± SD of 6 graft samples from each human Treg group, cotransferred with human PBMCs. *P* value (1-way ANOVA): **P < 0.01 and ****P < 0.0001.

To determine whether the potency shown by HLA-DR^+^CD27^+^ DP-enriched Xn-Tregs in suppressing islet xenograft rejection was associated with an increased accumulation of FOXP3^+^ Tregs within the graft, we performed a triple immunofluorescence staining of NICC xenografts with anti-human CD4 and FOXP3 antibodies and anti-porcine insulin antibody to look simultaneously for the presence of intact NICC grafts and intragraft human CD4^+^ T cells coexpressing FOXP3. Consistent with the results seen in Figure 5, HLA-DR^+^CD27^+^ DP-enriched Treg cotransferred mice had large and intact insulin-secreting NICC xenografts (Figure 6A). Associated with these intact functioning islet xenografts was an increased number of CD4-positive-staining cells coexpressing FOXP3 and surrounding the intact islet xenografts (Figure 6A). In mice cotransferred with human PBMCs plus Xn-Tregs, Pc-Tregs, or DP-depleted Xn-Tregs, only small fragments of NICC xenografts or single islet cells were seen. As expected, there were fewer intragraft FOXP3^+^CD4^+^ cells found in these mice (Figure 6A). The proportion of intragraft CD4^+^FOXP3^+^ cells was further analyzed by flow cytometry. The results revealed that 20.6% ± 5.5% of intragraft CD4^+^ T cells in HLA-DR^+^CD27^+^ Treg cotransferred mice coexpressed FOXP3 (Figure 5A), and this CD4^+^FOXP3^+^ cell proportion was significantly higher than that found in mice transferred with other Treg subsets (20.9% ± 2.8% vs. 10.5% ± 2.9% vs. 8.0% ± 3.6% vs. 4.4% ± 1.9%, for DP-enriched Xn-Treg vs. Xn-Treg vs. Pc-Treg vs. DP-depleted Xn-Treg, respectively) (Figure 6B). These results suggest that more potent suppression of islet xenografts in HLA-DR^+^CD27^+^ DP-enriched Xn-Treg cotransferred mice was associated with a larger intragraft accumulation of functioning Tregs, which were functionally stable with in vitro and in vivo evidence of antigen specificity.

### Adoptive transfer of HLA-DR^+^CD27^+^ DP-enriched Xn-Tregs and PBMCs lead to a gene profile consistent with enhanced Treg function within islet xenografts.

**Figure 7:**
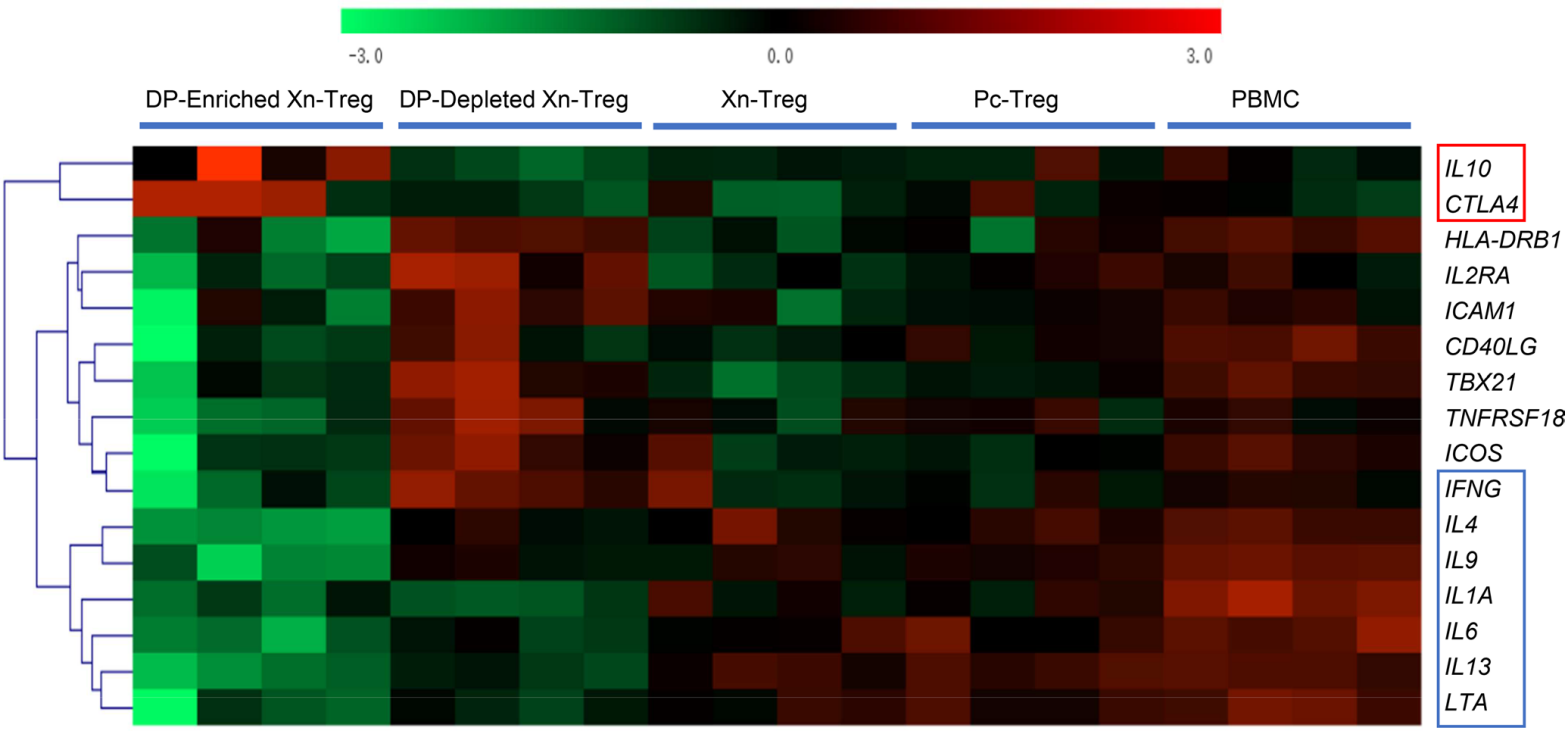
Examination of intragraft immune gene profiles in humanized recipients of NICC grafts. The heatmap represents normalized and color-coded relative expression values of differentially expressed genes (log_2_FC >2.0; FDR < 0.05, HLA-DR^+^CD27^+^ DP-enriched Xn-Treg vs. PBMC) (*n* ≥ 4 individual mice from each group) in islet xenografts from recipient mice cotransferred with PBMCs and different Treg subsets at day 60 and the PBMC alone rechallenge group at day 35. The genes relating to Treg and T effector cell function are shown in the red and blue boxes, respectively. The distance metric is based on Euclidean distance. Red values indicate upregulated, and green values indicate downregulated.

To determine if grafts protected by the infusion of HLA-DR^+^CD27^+^ DP-enriched Xn-Treg had a distinctive antiinflammatory genetic profile compared with grafts taken from mice infused with other Treg populations, NICC grafts were taken from recipient mice at 60 days after PBMC/Treg transfer and at 35 days in recipient mice that received PBMCs alone, and intragraft RNA was evaluated using the TaqMan human immune panel consisting of 96 target genes from immune system functions, including those associated with Treg and effector T cell function. Differentially expressed genes in surviving islet xenografts from mice receiving HLA-DR^+^CD27^+^ DP-enriched Xn-Tregs were compared with rejecting xenografts from mice that received human PBMCs alone. The heatmap displayed a picture of predominant Treg function from the islet xenografts of mice that received PBMCs and HLA-DR^+^CD27^+^ DP-enriched Tregs, with downregulated expression of effector Th1, Th2, and Th17 cytokine genes along with elevated intragraft expression levels of 2 key Treg function genes, *IL10* and *CTLA4* (Figure 7). By contrast, in the rejected NICC grafts from mice given PBMCs alone or from mice cotransferred with any other Treg subset, there were expression of comparatively low levels of *IL10* and *CTLA4* and higher levels of gene expression of all effector T cell cytokines examined (Figure 7). In addition to the differences in the *IL10* and *CTLA4* gene expression, there were significant differences in gene expression associated with other functional T cell subsets among the different groups of mice, and these results were shown in Table 1. Compared with mice receiving both human PBMCs and Pc-Tregs or Xn-Tregs or DP-depleted Xn-Tregs, mice cotransferred with human PBMCs and HLA-DR^+^CD27^+^ DP-enriched Tregs had the largest downregulation of effector Th1 (*IFNG* and *LTA*), Th2 (*IL4*, *IL9*, and *IL13*), and Th17 (*IL17*) cytokine gene expression, as well as the greatest upregulation of Treg-associated gene expression (*IL10* and *CTLA4*) (Table 1). This verifies the more powerful suppressive function of the HLA-DR^+^CD27^+^ DP-enriched Xn-Treg subset and the subset’s resistance to being converted to effector Th cells in the presence of an inflammatory response. Taken together, these findings suggest that HLA-DR^+^CD27^+^ DP-enriched Xn-Tregs were able to selectively target the NICC graft and exert an effective and stable suppressive function that regulates the human effector Th cell–initiated xenogeneic immune response in vivo.

## Discussion

Given their role in regulating immunity, infusion of Tregs after ex vivo manipulation has been proposed as a treatment for autoimmune diseases and/or tolerance induction in transplantation. Numerous preclinical studies have indicated that antigen-specific Treg cells are more potent than polyclonal Treg cells in the control of immune responses in autoimmune diseases and transplantation (6). They have the ability to migrate toward the site of antigen presentation (30), thereby leading to more efficient and localized control of inflammation without the risks of broad immunosuppression and associated adverse events. The enhanced trafficking of antigen-specific Tregs to target tissues may allow the administration of fewer Tregs than currently used in trials of polyclonal Tregs, which should allow for more efficient and cost-effective in vitro expansion protocols for Treg adoptive cell therapy. Generation of antigen-specific Tregs in vitro has been reported by induction of antigen-specific effector T cells into cells with suppressive capacity (31, 32), expansion of Tregs with allogenic DCs or B cells (16, 33), or genetic engineering of TCR- or CAR-Tregs (17, 34–36). However, as with polyclonal Tregs, antigen-specific Tregs will need to demonstrate prolonged survival, stability, and lack of plasticity in vivo (37).

In this study, we described a simple and effective approach to produce antigen-specific Tregs that meet the requirements for adoptive Treg therapy. By culturing naive/resting human Tregs in the presence of porcine PBMCs we selectively expanded a distinct subset of Tregs coexpressing activation/memory surface markers HLA-DR and CD27. We show that compared with their unsorted and HLA-DR and CD27 DP cell–depleted counterparts, HLA-DR^+^CD27^+^ DP-enriched Xn-Tregs were antigen specific, with enhanced suppression of the xenogeneic response in association with upregulated expression of Treg function markers CD95 and ICOS. Moreover, HLA-DR^+^CD27^+^ DP-enriched Treg are more stable in their phenotype and function and are resistant to conversion to effector Th1 and Th17 cells under inflammatory conditions. This is confirmed by their demethylation TSDR state and high level of Helios expression that was similar to that of unexpanded naive/resting Tregs. Adoptive transfer of porcine islet graft recipient NSG mice with even a small number of HLA-DR^+^CD27^+^ DP-enriched Xn-Tregs efficiently inhibited porcine islet graft rejection mediated by 25-fold more human effector cells in a humanized mouse model. The prolonged graft survival was associated with downregulated intragraft expression of effector Th1, Th2, and Th17 cytokine genes; enhanced accumulation of FOXP3^+^ Tregs within surviving grafts; and upregulated intragraft expression of Treg functional genes *IL10* and *CTLA4*. These findings verified the functional stability and the capacity of adoptively transferred HLA-DR^+^CD27^+^ DP-enriched Tregs to migrate to the site of the transplanted graft. In previous studies using unselected expanded human Tregs in higher doses, we demonstrated effective suppression of porcine islet xenograft rejection and GVHD in NSG recipients reconstituted with human PBMCs (29). The observation that HLA-DR^+^CD27^+^ DP-enriched Xn-Tregs at much lower Treg/PBMC ratios could prevent porcine islet xenograft rejection without suppression of GVHD further highlights their enhanced antigen specificity.

Taken together, these findings indicate that in vitro antigen-stimulated HLA-DR^+^CD27^+^ DP-enriched Xn-Tregs acquired the properties of activated phenotype, potent suppression, and antigen specificity. CD27 is a member of the tumor necrosis factor (TNF) superfamily of costimulatory receptors associated with the regulation of immune responses. For example, CD27 expression has been shown to identify highly suppressive and antigen-specific Tregs, distinguishing them from activated CD25^+^CD4^+^ effectors (21, 38–41). Moreover, CD27 expression has been reported to be inversely correlated with Treg IL-17 production in the skin of patients with psoriasis and hidradenitis suppurativa, suggesting the role of CD27 in regulating Treg plasticity in inflammatory tissue (42). HLA-DR expression has been associated with active rather than resting Tregs, and HLA-DR^+^ Tregs are more suppressive than HLA-DR^–^ counterparts (20, 24). Studies have also shown that alloantigen-induced regulatory CD3^+^CD4^+^HLA-DR^+^ T cells express the regulatory and activation markers CD25, CTLA4, CD62L, PD1, and TNFRII (43). Autologous PBMC-stimulated CD8^+^HLA-DR^+^ Tregs revealed similar phenotypic and functional features to CD4^+^FOXP3^+^ Tregs, with highly antigen-specific suppression of responder CD8^+^ T cells (44). Thus, coexpression of HLA-DR and CD27 allows us to identify and sort a highly suppressive and antigen-specific Treg subset with stable features from unstable subsets in xenoantigen-expanded human Treg populations. HLA-DR^+^CD27^+^ Tregs that are expanded by xenoantigen stimulation have the potential to meet the proposed criterion of potent suppressive activity, antigen specificity, functional stability, and capacity to localize at the site of the transplanted graft. However, it should be noted that identification of a molecular signature and profiling of TCR clonotypes in HLA-DR^+^CD27^+^ Tregs would help us further characterize their antigen specificity and functional stability.

Tregs mediate their immunosuppressive effects via a variety of mechanisms, including immunosuppressive cytokines, such as IL-10, TGF-β, and IL-35; consumption of IL-2; production of immunosuppressive adenosine by ectoenzymes CD39 and CD73; modulation of antigen-presenting cells by CTLA4, LAG3, and ICOS; and granzyme- and perforin-mediated cytolysis (45–49). Tregs can also target effector T cells directly by Fas/Fas-mediated apoptosis (28, 50, 51). Moreover, the Treg activation/memory marker ICOS (23, 24) not only stabilizes FOXP3 function but also increases IL10 production by Tregs, thereby leading to enhanced Treg suppressive potency (52–56). In this study, HLA-DR^+^CD27^+^ DP-enriched Xn-Tregs were more suppressive than other Treg subpopulations, and this correlated with their upregulated expression of Fas and ICOS and a trend of increasing expression of CTLA4, FOXP3, and Helios, indicating the contribution of these suppressive mechanisms to their enhanced suppression potency in vitro. Moreover, islet xenograft recipients adoptively transferred with HLA-DR^+^CD27^+^ DP-enriched Xn-Tregs showed prolonged graft survival, while maintaining enhanced FOXP3 expression and elevated levels of *IL10* and *CTLA4*, further supporting the finding that HLA-DR^+^CD27^+^ DP-enriched Xn-Tregs retained their suppressive phenotype long term.

Although this study demonstrates the potential of in vitro antigen-primed HLA-DR^+^CD27^+^ Tregs to suppress T cell–mediated effector function, its findings should be interpreted within the limitations of the model. Although HLA-DR^+^CD27^+^ DP-enriched Xn-Tregs had enhanced expression of CD95 and ICOS and increased proportion of FOXP3^+^Helios^+^ Tregs, the precise mechanisms for the enhanced suppression remain unclear. Although their enhanced regulatory function and antigen specificity were verified in vivo after their infusion in NSG recipients of porcine islet grafts reconstituted with human PBMCs, this model does not result in full immune reconstitution. In particular, there are limited B cell and NK cell populations. While these cell populations can contribute to the rejection response, B cells have also been implicated in the development of tolerance in both mouse and human studies, making the in vivo findings here even more remarkable (57). Despite these limitations, this study has identified a subpopulation of Treg cells that are enriched for antigen-specific Tregs with enhanced suppressive function. Upon in vitro expansion, HLA-DR^+^CD27^+^ expression emerges for the first time to our knowledge as a specific Treg activation signature allowing the identification and isolation of an epigenetically stable antigen-activated Treg subset, provides the means and essential knowledge for the design of improved Treg-base cell therapy, and could form the basis for more personalized therapy in immunosuppression.

## Methods

### Animals.

Newborn and adult Landrace pigs from local farms in New South Wales, Australia, were used as donors for NICCs and PBMCs, respectively. NSG mice obtained from Australia BioResources were housed under specific pathogen–free conditions in the Biological Services Facility of The Westmead Institute for Medical Research. Mice between the ages of 6–8 weeks were used for NICC transplantation.

### PBMC isolation and expansion of human Tregs.

Human PBMCs were obtained from healthy donor buffy coat samples provided by NSW Red Cross, Australia. CD4^+^CD25^+^CD127^–/lo^ Tregs were isolated from PBMCs as described previously (18). Porcine PBMCs from Landrace pigs and human PBMCs from a single donor were used as xenogeneic and allogeneic stimulator cells, respectively. The resulting CD4^+^CD25^+^CD127^–/lo^ Tregs were cultured in 96-well, round-bottom plates (5 × 10^4^/well) in RPMI 1640 medium (Gibco), supplemented with 10% human AB serum (Invitrogen), 2 mmol/L glutamine, 25 mmol/L HEPES, 50 U/mL penicillin, 50 μg/mL streptomycin, 50 μM 2-mercaptoethanol (MilliporeSigma), and 100 nmol/L rapamycin (MilliporeSigma) at 37°C and 5% CO_2_, in the presence of 400 U/mL IL-2 (Chiron) either stimulated with anti-CD3/CD28 dynabeads (Invitrogen) at a 1:1 ratio of Treg/bead and designated as Pc-Treg, or stimulated with anti-CD3/CD28 dynabeads combined with irradiated porcine PBMCs (30 Gy) at a 4:1 ratio of porcine PBMCs/Tregs (4 × 10^5^ porcine PBMCs: 1 × 10^5^ Tregs, Xn-Treg), respectively. After 7 days, cells were counted and split every 3 days; supplied with fresh RPMI 1640 medium containing IL-2, rapamycin, and anti-CD3/CD28 dynabeads; and restimulated with irradiated pig PBMCs at days 7 and 14 as described above. Expanded Tregs were continuously counted at days 14 and 21 and harvested at day 21 for subsequent experiments (18).

Al-Tregs were produced from CD4^+^CD25^+^CD127^–/lo^ Tregs, isolated from PBMCs, and expanded in the presence of irradiated alloantigen PBMCs, as described for the production of Xn-Tregs.

### Flow cytometry and cell sorting.

Flow cytometric analysis of Treg phenotype, human leukocyte engraftment, and graft-infiltrating human leukocytes was performed as described previously (18, 29) by staining in different combinations with the following antibodies according to manufacturer’s recommendations: CD4-APC-H7, CD4-PE-Cy7, CD127-PE, CD27-BV711, HLA-DR-FITC, CD62L-PE, 7AAD, CTLA-4-PE, ICOS-BV421, IL-17A-BV421, IFN-γ-BV711, and Foxp3-PECF594 from BD Biosciences; CD25-APC and Foxp3-PE from eBioscience; CTLA-4-BV605, Helios-Pacific blue, and CD95-BV421 from BioLegend; and GITR-PE from R&D Systems. Intracellular cytokine staining was performed using the BD Cytofix/Cytoperm Fixation and Permeabilization Solution, and intracellular staining for FOXP3, Helios, and CD95 was performed using the Foxp3 Buffer Set from eBioscience, all according to manufacturers’ protocols. The used antibodies are detailed in Supplemental Table 1. All data were acquired on LSR II, LSR Fortessa, or Symphony (BD Biosciences) and analyzed using FlowJo (TreeStar). Cell sorting was performed on a FACSAria III (BD Biosciences) by the following gating strategy. Xn-Tregs and Al-Tregs stained with CD4-APC-H7, CD25-APC, CD127-PE, CD27-BV711, HLA-DR-FITC, and 7AAD were first gated on human lymphocytes and 7AAD^–^ alive cells to deplete residual irradiated porcine cells and dead cells, followed by gating on CD4^+^ cells, then CD25^+^CD127^–^ cells (Supplemental Figure 4), which were finally sorted into HLA-DR^+^CD27^+^ DP-enriched Xn-Treg and DP-depleted Xn-Treg subsets, respectively. Pc-Tregs also went through the cell sorter to deplete dead cells. The purities of the resulting HLA-DR^+^CD27^+^ DP-enriched Xn-Treg and DP-depleted Xn-Treg subsets were all greater than 98%, respectively. The isolated cells were used after an overnight rest at 37°C in the same Treg culture medium as used during expansion.

### Suppression assays.

The suppressive capacity of Treg was assessed by MLR assays. CFSE-labeled (Invitrogen) (1 **×** 10^5^) responder cells (autologous PBMCs without CD4^+^CD25^+^CD127^–^ Tregs) were either incubated with anti-CD3/CD28 dynabeads at a 1:3 ratio of cells/beads for polyclonally stimulated MLR assays (Poly MLR) or cocultured with 3 **×** 10^5^ irradiated (30 Gy) xenogeneic or allogeneic PBMCs for xenogeneic (Xeno MLR) or allogeneic (Allo MLR) MLR, respectively. Tregs were titrated into different MLR cultures at different ratios. After 3 days of culture for Poly MLR, or 7 days for Xeno and Allo MLR, proliferation of responder cells (CFSE-positive cells) was evaluated based on the percentage of proliferating responder cells cultured in the absence of Tregs compared with the percentage of proliferating responder cells cultured in the presence of Tregs. The percentage of proliferating responder cells in the absence of Tregs was considered as 100% of proliferation and 0% of suppression.

### Cytokine analysis.

IFN-γ was measured by ELISA (Human IFNγ ELISA Kit, Invitrogen) in supernatants collected from the above Xeno MLR assays according to the manufacturer’s recommendation.

### TSDR analysis.

DNA samples, used for TSDR detection, were extracted from Fresh-Tregs (no expansion), Pc-Tregs, Xn-Tregs, HLA-DR^+^CD27^+^ DP-enriched Xn-Tregs, and DP-depleted Xn-Tregs, with AllPrep DNA/RNA Mini Kit (QIAGEN). Purified DNA (200 ng) was subjected to sodium bisulfite conversion with an EZ DNA Methylation-Gold Kit (Zymo Research). For detection of methylated CpGs, bisulfite converted genomic DNA was subjected to quantitative PCR (qPCR) using HEX- and FAM-labeled probes that recognize methylated and demethylated CpG sites, respectively. The methylation qPCR 5′ primer is ATTTGGGTTTTGTTGTTATAGTTTT and 3′ primer is AAAATATCTACCCTCTTCTCTTCCTC. The probes include 6FAM/Zen-ATGGTGGTTGGATGTGTTGGGTT-lBFQ and HEX/Zen-ATGGCGGTCGGATGCGTC-lBFQ. Methylation was calculated using the formula: %methylation = 100/(1 + 2^Ct[methylated^^–^^unmethylated]^) (58).

### Inflammatory stimulation.

Tregs were collected after sorting and placed immediately into culture media — 90% RPMI 1640, 10% FBS (Invitrogen), and 50 mM 2-mercaptoethanol (MilliporeSigma), 100 nM rapamycin (MilliporeSigma) containing 10 IU/mL IL-2, 10 ng/mL IL-1β, 5 ng/mL IL-6, 25 ng/mL IL-21, 25 ng/mL IL-23, and 5 ng/mL TGF-β (all cytokines from R&D Systems) — for 6 days for inflammatory cytokine-producing cell induction. Cells cultured in the same medium containing 10 IU/mL IL-2 only were used as noninduction controls (nonstimulated). After 6 days of stimulation, cells were further incubated with 50 ng/mL phorbol 12-myristate 13-acetate (MilliporeSigma), 1 μg/mL ionomycin (MilliporeSigma), and GolgiStop protein transport inhibitor (BD Biosciences) for a further 5 hours prior to cytometric analysis of percentage of CD4^+^ cells coexpressing IL-17 or IFN-γ, respectively.

### Porcine islet isolation and transplantation.

NICCs were isolated from the pancreases of piglets (1–3 days old) and propagated in culture for 6 days as described previously (59). A total of 4,000 NICC islet equivalents were transplanted into NSG mice under the renal capsule of both kidneys.

### Adoptive transfer of human cells.

A total of 1 × 10^7^ human PBMCs that were depleted of CD4^+^CD25^+^CD127^–/lo^ Tregs were injected i.v. alone or together with 4 × 10^5^ ex vivo–expanded autologous Treg subsets into NSG mice 3 days after NICC transplantation. Peripheral blood, serum, spleen, and grafts were collected from recipient mice at predetermined time points after human cell transfer to assess human leukocyte engraftment and NICC xenograft survival. Graft rejection was defined as no visible intact graft observed by histological examination (29).

### Immunohistochemistry and immunofluorescence.

Histology and immunohistochemistry of cryostat sections (6 to 8 μm) and paraffin (4 μm) were undertaken as described previously (29). Porcine endocrine cells were detected using guinea pig anti-porcine insulin (Dako Laboratories), guinea pig anti-porcine glucagon (Linco Research), and goat anti-human somatostatin (Santa Cruz Biotechnology) antibodies and the Universal ABC Kit (Vector Laboratories). Graft-infiltrating human leucocytes were stained using rabbit or mouse anti-human CD4 (Abcam) and CD8 (Dako) antibodies, followed by incubation with horseradish peroxidase–conjugated secondary goat anti-mouse antibody (Abcam). Sections were visualized with diaminobenzidine (Dako). Triple immunofluorescence staining of human CD4 and FOXP3 and porcine insulin was undertaken with rabbit anti-human CD4 polyclone Ab and mouse anti-human Foxp3 mAb (Abcam) and guinea pig anti-porcine insulin, followed by secondary incubation with Alexa Fluor 488–conjugated goat anti-mouse Abs and Alexa Fluor 562–conjugated goat anti-rabbit (Abcam). The sections were then stained with DAPI (MilliporeSigma). The used antibodies are detailed in Supplemental Table 2. The sections were viewed under an Olympus FV1000.

### Porcine C-peptide assay.

Porcine C-peptide in NICC recipient mouse serum was measured using Mercodia Porcine C-peptide ELISA kit (Mercodia AB) according to the manufacturer’s instructions in a Victor X3 multilabel plate reader (PerkinElmer).

### TaqMan human immune panel array.

Islet xenografts were harvested from both kidneys of recipient mice at predetermined time points as indicated. One graft from each mouse was fixed in 4% formalin for histological studies, and the other one was cryopreserved for immunohistology and RNA extraction using the RNA extraction kit (QIAGEN). RNA quality and quantity were determined using Nano Drop (Thermo Fisher Scientific). RNA was reverse-transcribed into cDNA using SuperScript IV VILO Master Mix with ezDNase enzyme (QIAGEN). Gene expression assay was performed using TaqMan Human Immune Panel (Applied Biosystems) according to the manufacturer’s instruction by QuantStudio 12K Flex Real-Time PCR System (Thermo Fisher Scientific). The mean expression of GUSB and GAPDH was used for normalization, and the results were analyzed by ExpressionSuite software (Thermo Fisher Scientific). The genes of interest and differentially expressed genes were selected using the volcano plot for PBMCs versus HLA-DR^+^CD27^+^ DP-enriched Xn-Tregs comparison with FDR < 0.05 and a greater than a 2-fold change. Heatmaps were generated with Multiple Experiment Viewer software (https://sourceforge.net/projects/mev-tm4/, version MeV4.9.0), and unsupervised hierarchical clustering was performed using Euclidean correlation.

### Statistics.

Results involving multiple groups were evaluated using 1-way or 2-way ANOVA with Tukey’s multiple-comparison test or Kruskal-Wallis multiple-comparison test (nonparametric) (GraphPad Prism version 8.0). For differences between 2 Treg expansion protocols or 2 types of Treg subsets, the paired-comparison *t* test (2 tailed) was used. Data were presented as mean ± SD for all except MFI data that were presented as mean ± SEM. *P* less than 0.05 was considered statistically significant.

### Study approval.

The study was approved by the Westmead Area Health Service Human and Animal Ethics Committees and conducted in compliance with State Government Legislation and National Health and Medical Research Council Animal Research Guidelines.

### Data availability.

All data needed to evaluate the conclusions in the paper are present in the paper or the Supporting Data Values file. Any additional information required to reanalyze the data reported in this paper is available upon request.

## Author contributions

XM, LC, and MR as co–first authors performed and interpreted the results of experiments, analyzed data, prepared figures, and drafted the manuscript. XM was responsible for the establishment of the transplant model and related experiments, and her name appears first because of her role in the initiation of the project; LC and MR were responsible for the phenotypic and functional experiments in vitro. QC performed animal experiments and analyzed data. HW and YZ performed some Treg isolation and flow cytometric analysis experiments. NGB and GN performed TSDR analysis. LCH reviewed and revised the manuscript. WJH isolated and provided pig islets and performed pig-to-mouse islet transplantation experiments. MH designed the research, interpreted the results of experiments, analyzed data, edited the manuscript, and finalized the revised version of the manuscript. SY and PJO conceived and designed the research, interpreted the results of experiments, analyzed data, and revised the manuscript. All authors approved the final version of the manuscript. PJO is the guarantor of this work. MH, SY, and PJO had full access to all the data in the study and take responsibility for the integrity of the data and the accuracy of the data analysis.

## Supplementary Material

Supplemental data

Supporting data values

## Figures and Tables

**Table 1:**
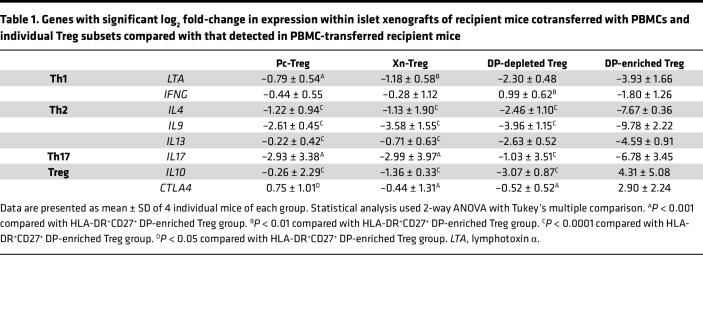
Genes with significant log_2_ fold-change in expression within islet xenografts of recipient mice cotransferred with PBMCs and individual Treg subsets compared with that detected in PBMC-transferred recipient mice

## Version 1 10/24/2023

In-Press Preview

## Version 2 12/08/2023

Electronic publication
