# Supplementary material for: Human HLA-DR+CD27+ regulatory T cells show enhanced antigen-specific suppressive function
Source: JCI Insight. 2023 Dec 8;8(23):e162978. doi: 10.1172/jci.insight.162978 (PMC10795828; doi:10.1172/jci.insight.162978)
Supplement: Supplemental data [file jciinsight-8-162978-s021.pdf]

A

Marker	CD4	CD25	FOXP3	CD127	HLA-DR	CD3	CD27	Helios
**Fluorochrome**	APC-H7	APC	PE-CF594	PE	FITC	PerCP	BV711	Pacific Blue
**Laser (nM)**	637	637	561	561	488	488	406	406
**Power (mw)**	140	140	150	150	200/150	200/150	200	200
**Detector**	R820_60	R670_30	Y610_20	Y586_15	B515_30	B670_30	V710_50	V474_25

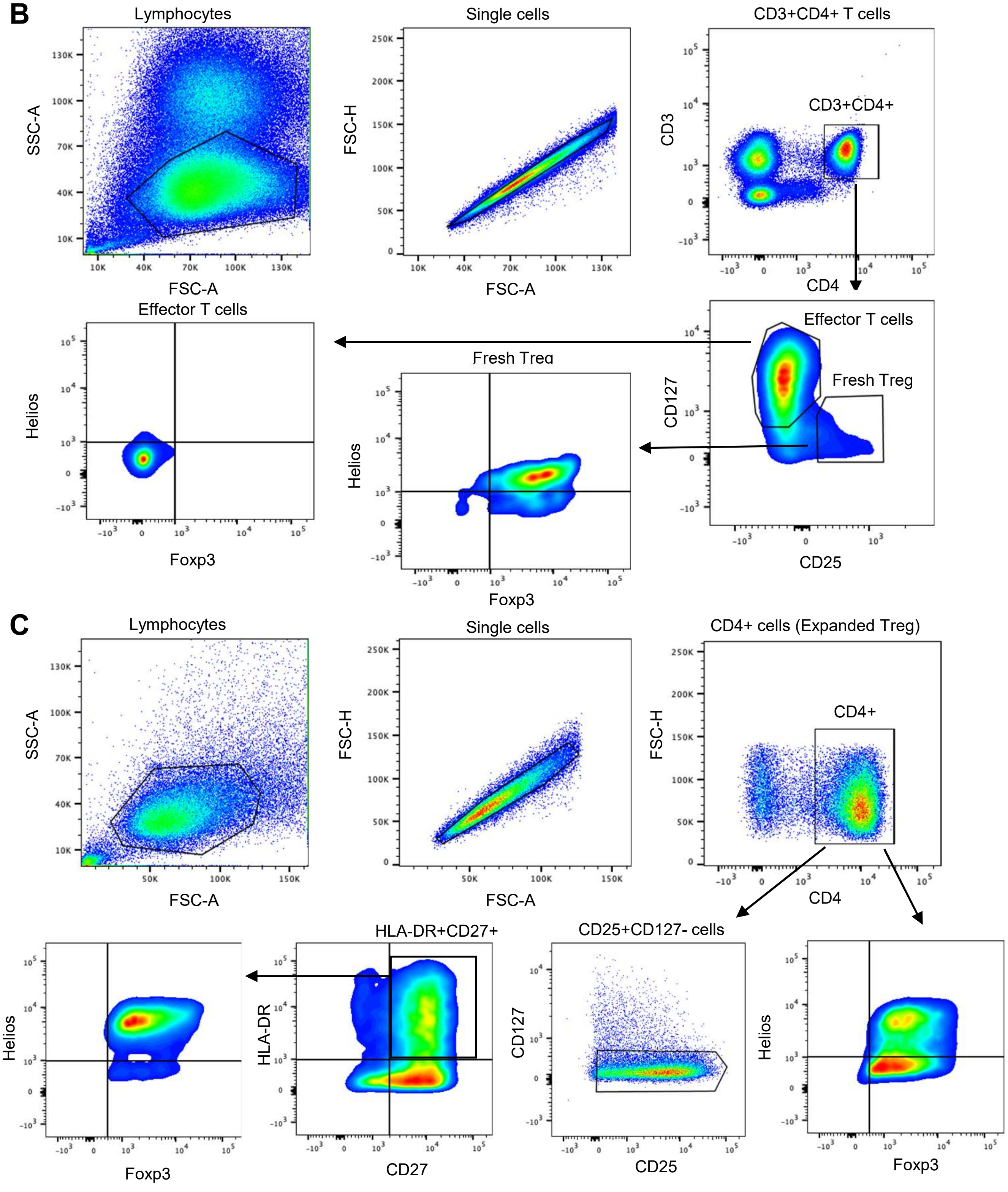

Supplemental Figure 1. Gating strategies for FOXP3 and Helios co-expression. (A) Multicolor flow cytometry panel performed on BD FACSymphony Cell Analyzer for phenotypic analysis of co-expression of FOXP3 and Helios on different types of Treg. **(B)** Gating strategies for FOXP3 and Helios co-expression on effector T cells and Fresh-Tregs and **(C)** polyclonal stimulated Tregs (Pc-Treg), xenoantigen expanded Treg (Xn-Treg) and HLA-DR+CD27+ DP-Enriched Xn-Treg. Series of gates used to distinguish effector T cells and Fresh-Tregs from human PBMC for identifying the proportion of FOXP3+Helios+ cells. These are the gates for lymphocyte proportion, excluding doublets and CD4+CD3+ T cells, then CD25-CD127+/low and CD25+CD127-/low on CD25 vs. CD127 flow cytometric plots *(B)* (data shown in Figure 1B). After three stimulation rounds, ex-vivo expanded Xn-Tregs and Pc-Tregs were gated on CD4+ and the proportion of FOXP3+Helios+ cells on Pc-Treg and Xn-Treg was assessed *(C)* (data shown in Figure 1B). A further gate on HLA-DR vs. CD27 flow cytometric plots allowed the identification of FOXP3+Helios+ cells for HLADR+CD27+ DP-Enriched Xn-Treg (data shown in Figure 2C).

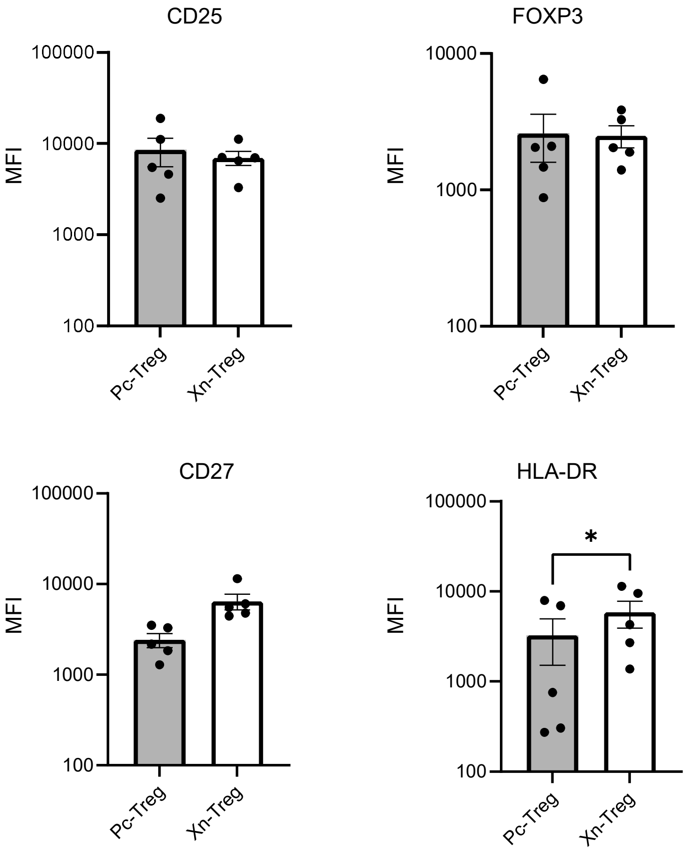

Supplemental Figure 2. Phenotypical characterization of Xn-Treg and Pc-Treg. Expression level (MFI) of CD25, FOXP3, CD27 and HLA-DR in polyclonal stimulated Tregs (Pc-Treg) and xenoantigen expanded Treg (Xn-Treg) after 3 rounds of stimulation. Data represented as mean ± SEM of three independent experiments with Treg from five individual donors. P value (Paired t test) (2-tailed): *P < 0.05.

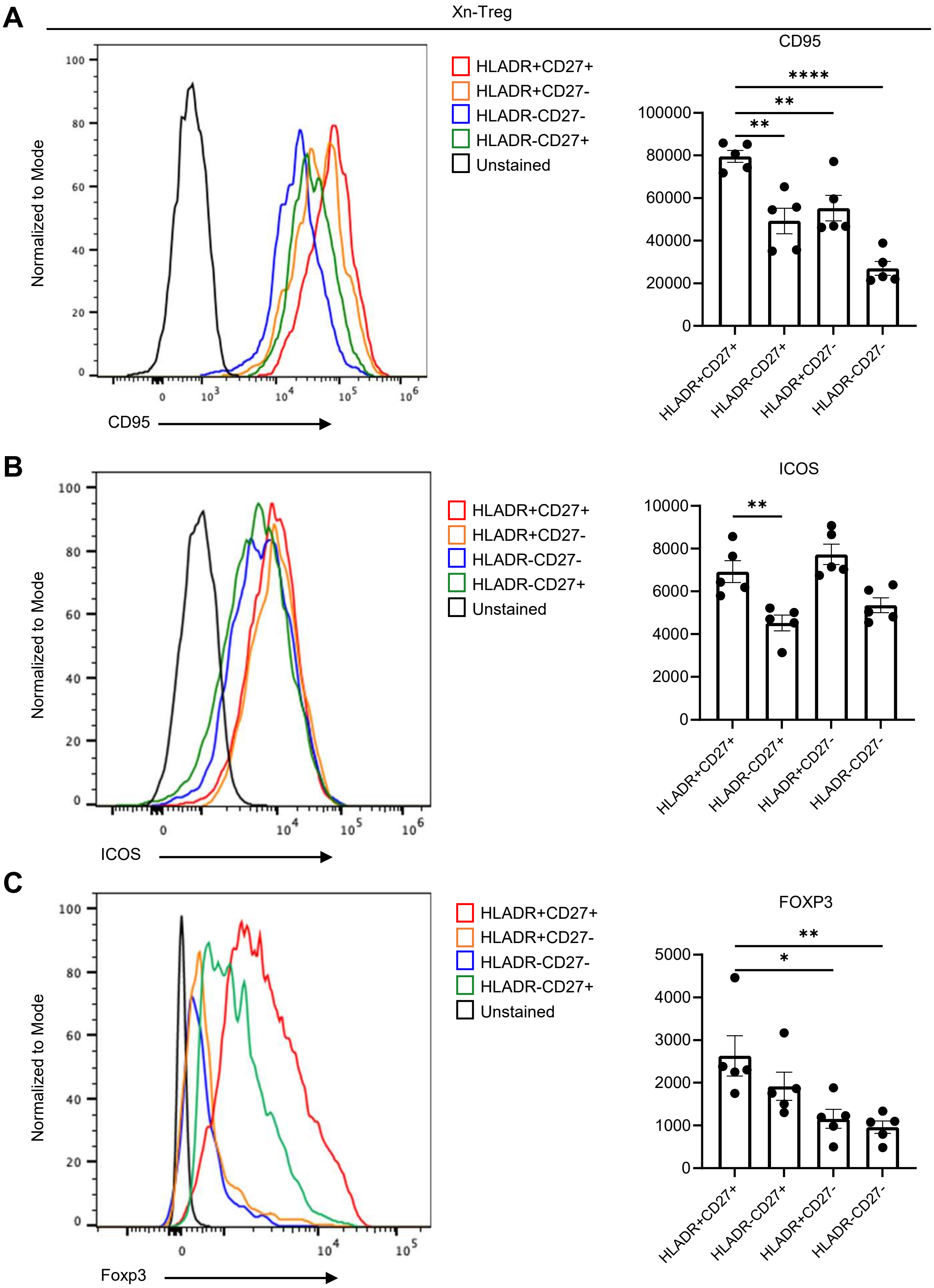

Supplemental Figure 3. The expression of CD95, ICOS, and FOXP3 was associated with HLA-DR and CD27 expression within Xn-Treg. Representative histograms of the expression level of CD95 **(A)**, ICOS **(B)** and FOXP3 **(C)** shown as MFI for four subpopulations within Xn-Treg, based on their expression of HLA-DR and/or CD27. Data represents three independent experiments with Xn-Treg from 5 individual donors. Error bars indicate the mean ± SEM. P value (1-way ANOVA): *P<0.05, **P< 0.01 and ****P< 0.0001.

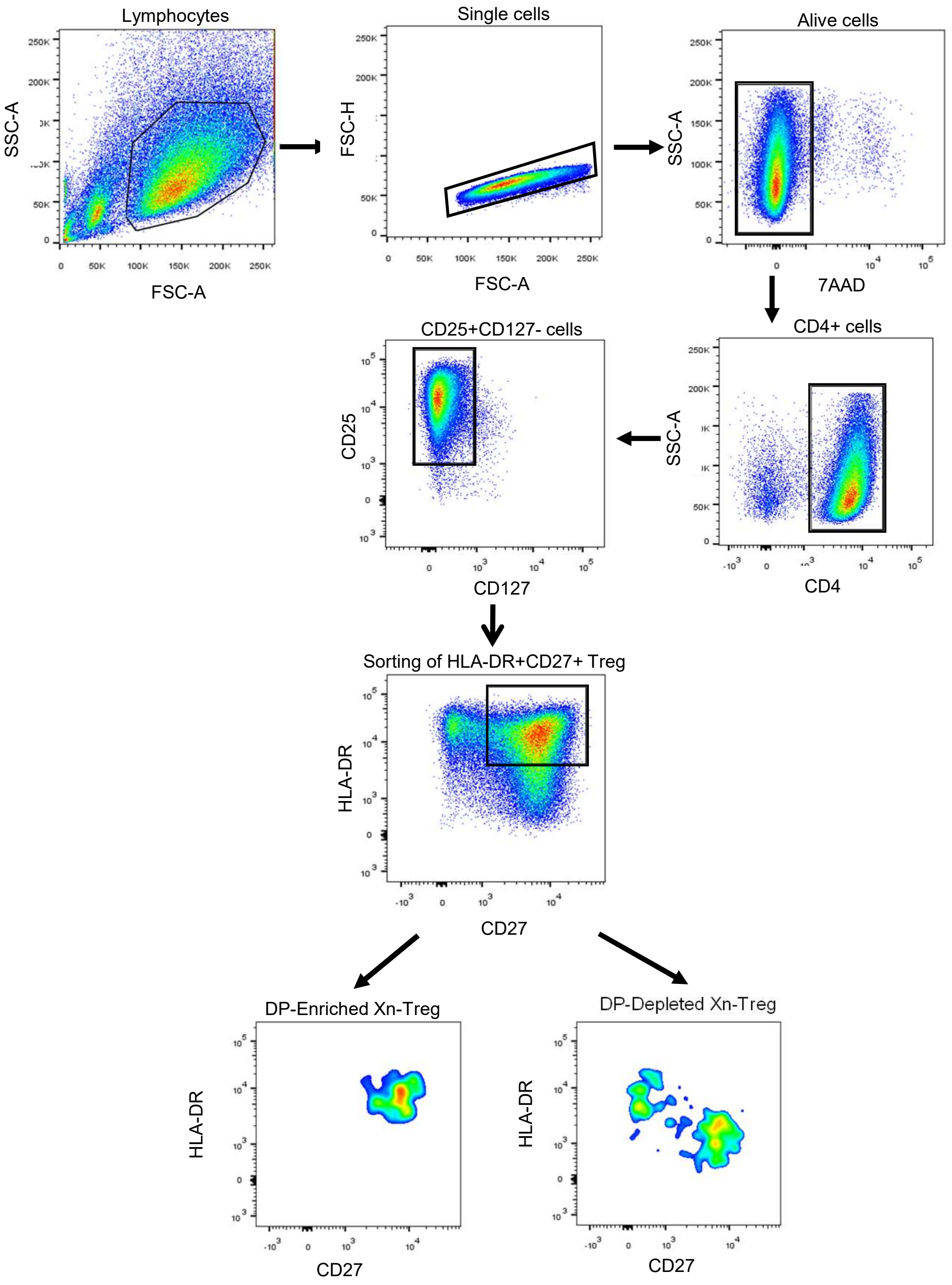

Supplemental Figure 4. Cell sorting strategy for isolation of HLA-DR+CD27+ DP Enriched Xn-Treg subset. After expansion with xenoantigen stimulation for three cycles, Xn-Treg were collected for cell sorting of HLA-DR+CD27+ DP-Enriched Treg. A series of cell gating was applied to deplete porcine cells, dead cells and CD4+CD25-CD127+ cells as indicated. The remaining cells (CD4+CD25+CD127-) were gated on HLA-DR+CD27+ cells to acquire HLA-DR+CD27+ DP-Enriched Xn-Treg and DP-Depleted Xn-Treg subsets.

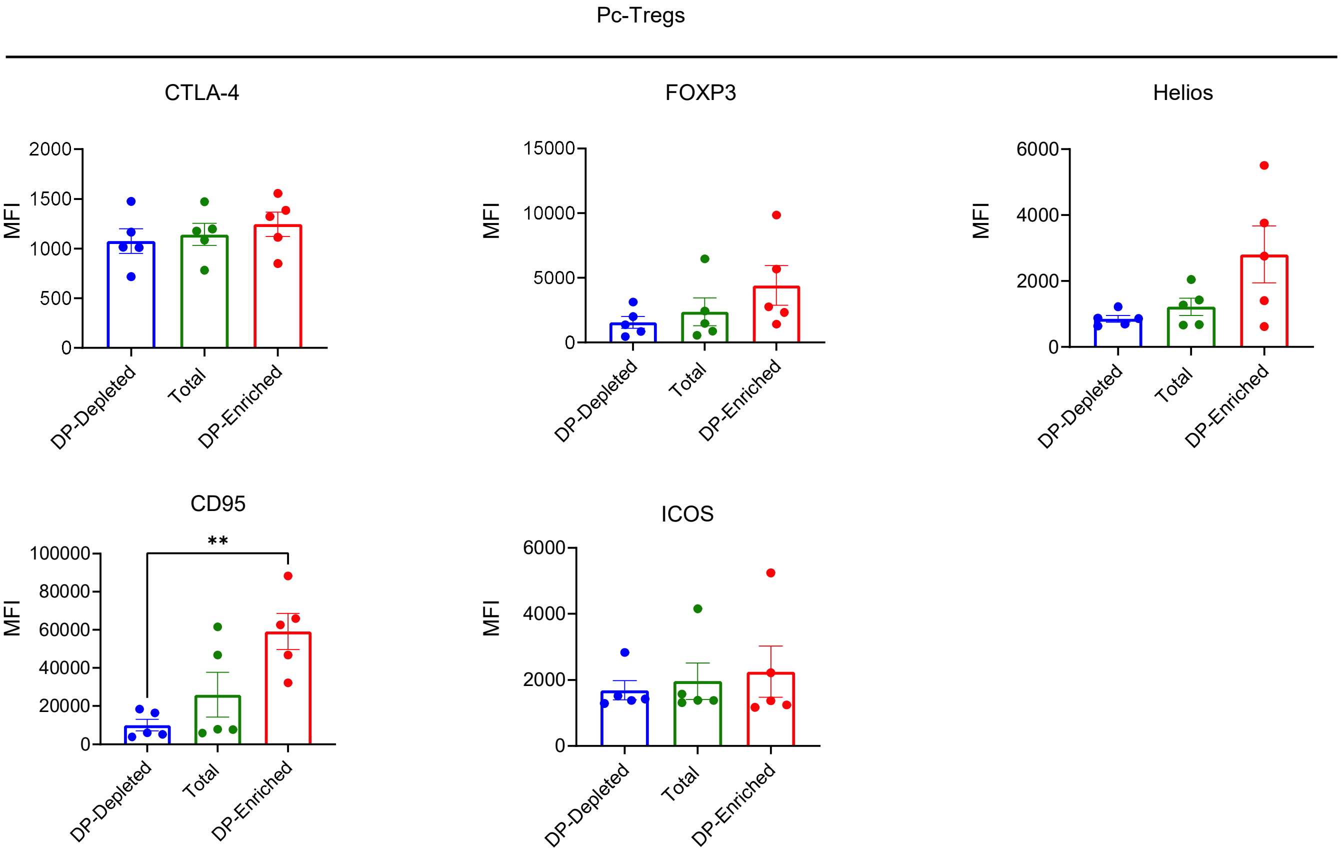

Supplemental Figure 5. Phenotyping of HLA-DR+CD27+ Treg subset analyzed on Pc-Treg. Expression level of CTLA-4 (surface), FOXP3 (intracellular), Helios (intracellular), CD95 (intracellular) and ICOS (surface) shown by the MFI in different types of Pc-Treg including total Pc-Treg (Total), HLA-DR+CD27+ DP-Enriched Pc-Treg (DP-Enriched) and DP-Depleted Pc-Treg (DP-Depleted). Data represents 3 independent experiments with Treg from 5 individual donors. P value (1-way ANOVA): **P< 0.01. Error bars indicate the mean ± SEM

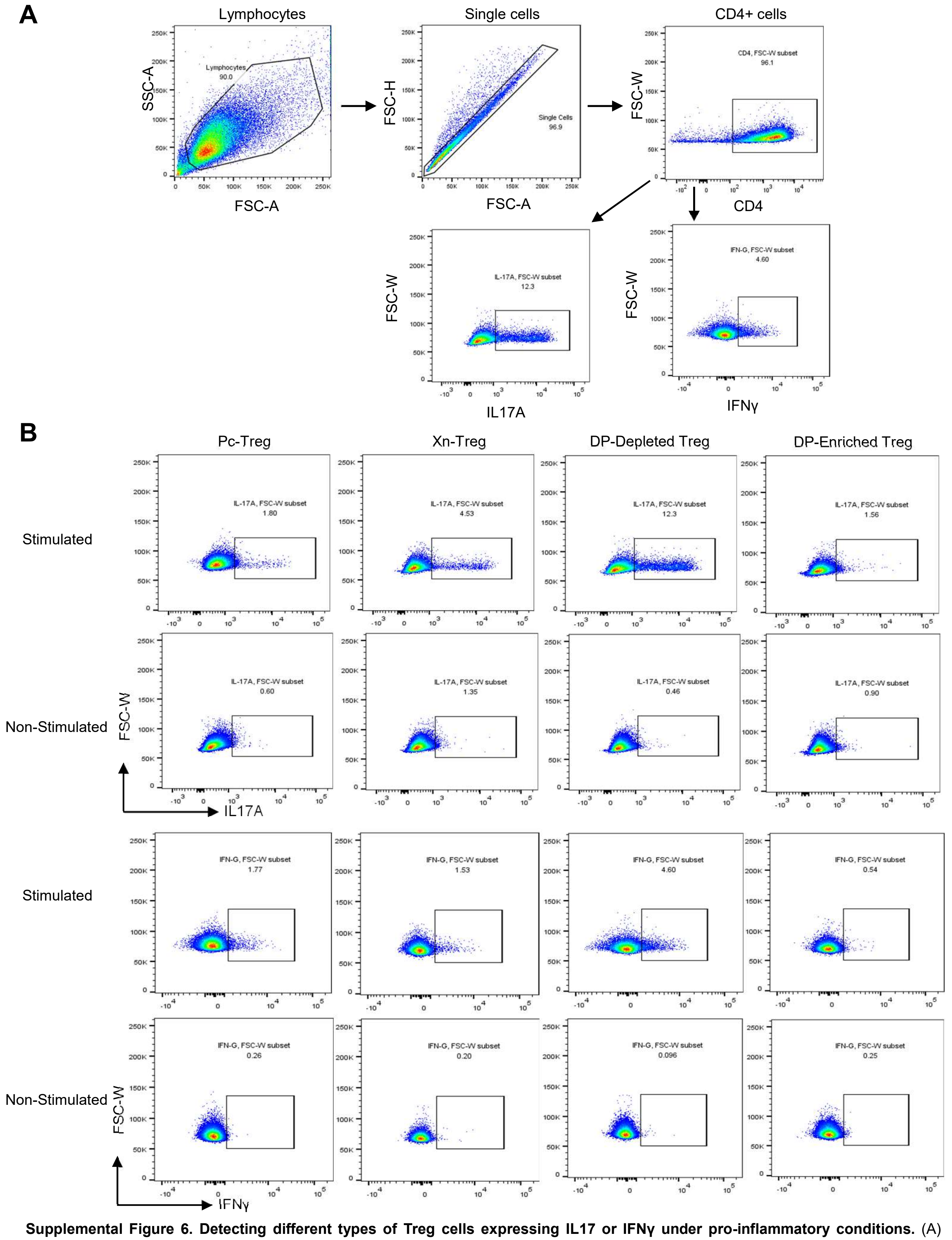

Supplemental Figure 6. Detecting different types of Treg cells expressing IL17 or IFNγ under pro-inflammatory conditions. (A) Gating strategy for detecting Treg cells expressing IL17 or IFNγ. A series of gates was applied including gates for lymphocyte proportion, excluding doublets then gated on CD4+ cells for further identifying the proportion of IL17 or IFNγ expressing Treg cells. (B) Representative flow cytometric plots showing the proportion of IL17 or IFNγ expressing Treg in different types of expanded Tregs under pro-inflammatory conditions. These types of Treg included Pc-Treg, HLA-DR+CD27+ DP-Enriched Xn-Treg and DP-Depleted Xn-Treg, and total Xn-Treg that were stimulated with a combination of pro-inflammatory cytokines (IL1β, IL6, IL21, IL23, TGFβ) and IL2 for 6 days (Stimulated). Control samples were Treg subsets with IL2 only (Non-Stimulated).

In Vivo Study Design

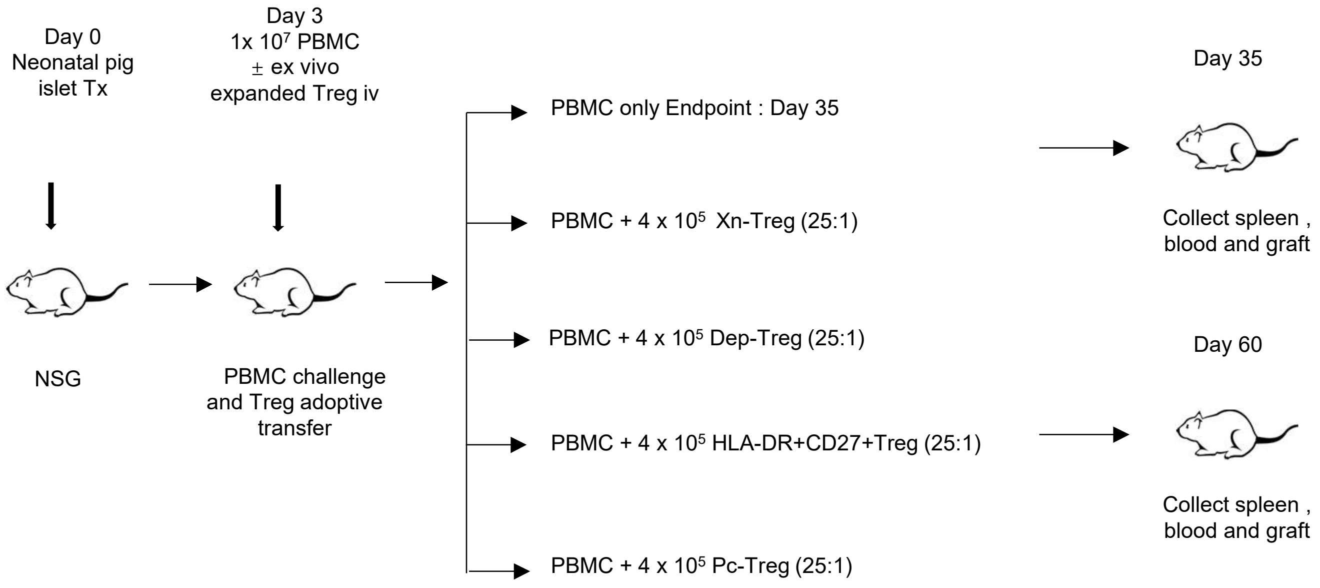

Supplemental Figure 7. A schematic representation of the in vivo humanized mouse model. NSG mice were transplanted with NICC xenografts and, 3 days after transplantation, were reconstituted with 10 × 7 human PBMC (CD4+CD25+CD127-/low Treg depleted) alone or PBMC co-transferred with 4 × 105 of different individual Treg subsets at a 25:1 ratio of PBMC:Treg. Blood, spleen and graft samples were taken at predetermined time points days 35 and 60 post transplantation or human cell transfer from recipient mice adoptively transferred with human PBMC alone or together with different individual Treg subsets, respectively for the subsequent experiments as performed in Figures 5, 6 and 7, and Table 1.

Supplemental Table 1. Antibodies for flow cytometric analysis and cell sorting

Antibodies Flow Cytometric Analysis and Cell sorting			
Antibody//Target	*Clone*	*Conjugate*	*Catalogue number, Supplier*
CD3	UCHT-1	PE	555333, BD Pharmingen
CD3	SP34-2	PerCP	552851, BD Pharmingen
CD4	RPA-T4	APC-H7	560158, BD Biosciences
CD4	SK3	PE-Cy7	348789, BD Pharmingen
CD8	RPA-T8	PE	555367, BD Pharmingen
CD25	BC96	APC	17-0259-42, eBioscience (Invitrogen)
CD27	L128	BV711	563167, BD Biosciences
CD39	TU66	BUV737	612852, BD Biosciences
CD45	2D1	FITC	347463, BD Biosciences
CD62L	DREG-56	BV650	563808, BD Horizon
CD62L	DREG-56	PE	555544, BD Pharmingen
CD95	DX2	BV421	305623, BioLegemd
CD127	hIL-7RM21	PE	557938, BD Pharmingen
CTLA-4 (CD152)	BN13(BNI3)	BV605	369609, BioLegend
CTLA-4 (CD152)	BNI3	PE	557301,BD Biosciences
ICOS (CD278)	DX29	BV421	562901, BD Horizon
FOXP3	259D/C7	PECF-594	562421, BD Biosciences
FOXP3	PCH101	PE	12-4776-42, eBioscience (Invitrogen)
GITR (CD357)	110416	PE	FAB689P, R&D systems
Helios	22F6	Pacific Blue	137210, BioLegend
HLA-DR	L243	FITC	347363, BD Pharmingen
IL-17A	N49-653	BV421	562933, BD Biosciences
IFN-γ	B27	BV711	564039, BD Horizon
7-AAD			559925, BD Pharmingen
Human Fc Block			564220, BD Pharmingen

Supplemental Table 2: Immunohistochemistry and immunofluorescence staining antibodies

**Primary Antibodies**		
Antibody/Clone	*Catalogue number*	*Supplier*
Guinea pig Anti-Insulin (polyclonal)	IR00261-2	Agilent Dako
Rabbit Anti-CD4 (polyclonal)	ab231460	Abcam
Mouse Anti-CD8 (C8/144B)	ab17147	Abcam
Mouse Anti-FOXP3 (236A/E7)	ab20034	Abcam
Guinea pig anti-porcine glucagon	LS-C202759	LS Bio
Goat anti-Somatostatin (polyclonal)	Sc-7819	Santa Cruz Biotechnology
**Secondary Antibodies and Counterstain**		
Rabbit Anti-Guinea pig immunoglobulins/HRP	P0141	Agilent Dako
Donkey ani-goat immunoglobulins/HRP	A15999	ThermoFisher
Goat Anti-Rabbit IgG H&L (Alexa Fluor 488)	Ab150081	Abcam
Goat Anti-Mouse IgG H&L (Alexa Fluor 594)	Ab150120	Abcam
Goat Anti-Guinea pig IgG H&L (Alexa Fluor 647)	Ab150187	Abcam
DAPI (Vectashield Mounting Medium)	H-1200-10	Vector Laboratories
